# Prevalent Exon-Intron Structural Changes in the *APETALA1*/*FRUITFULL, SEPALLATA, AGAMOUS-LIKE6*, and *FLOWERING LOCUS C* MADS-Box Gene Subfamilies Provide New Insights into Their Evolution

**DOI:** 10.3389/fpls.2016.00598

**Published:** 2016-05-02

**Authors:** Xianxian Yu, Xiaoshan Duan, Rui Zhang, Xuehao Fu, Lingling Ye, Hongzhi Kong, Guixia Xu, Hongyan Shan

**Affiliations:** ^1^State Key Laboratory of Systematic and Evolutionary Botany, Institute of Botany, Chinese Academy of SciencesBeijing, China; ^2^University of Chinese Academy of SciencesBeijing, China

**Keywords:** *APETALA1/FRUITFULL*, *SEPALLATA*, *AGAMOUS-LIKE6*, *FLOWERING LOCUS C*, exon-intron structural change

## Abstract

*AP1*/*FUL, SEP, AGL6*, and *FLC* subfamily genes play important roles in flower development. The phylogenetic relationships among them, however, have been controversial, which impedes our understanding of the origin and functional divergence of these genes. One possible reason for the controversy may be the problems caused by changes in the exon-intron structure of genes, which, according to recent studies, may generate non-homologous sites and hamper the homology-based sequence alignment. In this study, we first performed exon-by-exon alignments of these and three outgroup subfamilies (*SOC1, AG*, and *STK*). Phylogenetic trees reconstructed based on these matrices show improved resolution and better congruence with species phylogeny. In the context of these phylogenies, we traced evolutionary changes of exon-intron structures in each subfamily. We found that structural changes have occurred frequently following gene duplication and speciation events. Notably, exons 7 and 8 (if present) suffered more structural changes than others. With the knowledge of exon-intron structural changes, we generated more reasonable alignments containing all the focal subfamilies. The resulting trees showed that the *SEP* subfamily is sister to the monophyletic group formed by *AP1*/*FUL* and *FLC* subfamily genes and that the *AGL6* subfamily forms a sister group to the three abovementioned subfamilies. Based on this topology, we inferred the evolutionary history of exon-intron structural changes among different subfamilies. Particularly, we found that the eighth exon originated before the divergence of *AP1/FUL, FLC, SEP*, and *AGL6* subfamilies and degenerated in the ancestral *FLC*-like gene. These results provide new insights into the origin and evolution of the *AP1*/*FUL, FLC, SEP*, and *AGL6* subfamilies.

## Introduction

MADS-box genes encode a family of transcription factors that have been found in plants, animals, and fungi (Theissen et al., 2000; Becker and Theissen, 2003; Ferrario et al., 2004; Causier et al., 2010; Rijpkema et al., 2010). In plants, the best-studied MADS-box genes are those involved in the specification of floral meristem and floral organ identities. Protein products of these genes are characterized by existence of four regions: the MADS (M) domain, the intervening (I) region, the keratin-like (K) domain, and the C-terminal (C) region (Theissen et al., 1996; Nam et al., 2003). Extensive phylogenetic studies have revealed that these MADS-box genes belong to eight different subfamilies or lineages: *APETALA1* (*AP1*)/*FRUITFULL* (*FUL*), *APETALA3* (*AP3*), *PISTILLATA* (*PI*), *AGAMOUS* (*AG*), *SEEDSTICK* (S*TK*), *SEPALLATA1* (*SEP1*), *SEPALLATA3* (*SEP3*), and *AGAMOUS*-*LIKE6* (*AGL6*) (reviewed in Theissen et al., 2000; Becker and Theissen, 2003; Nam et al., 2003). Among these, the evolutionary histories of the *AP3, PI, AG*, and *STK* subfamilies are relatively clear and can be traced back to the most recent common ancestor (MRCA) of extant seed plants (Aoki et al., 2004; Kramer et al., 2004; Dreni and Kater, 2013; Dreni et al., 2013). The relationships among the remainder four subfamilies, however, are still controversial, although the sisterhood of *SEP1* and *SEP3* (collectively called *SEP*) has got consistent support. In some studies, *SEP* was resolved as the sister of *AP1*/*FUL* (Carlsbecker et al., 2003; Litt and Irish, 2003; Kim et al., 2005; Futamura et al., 2008; Li et al., 2010), whereas in others, it forms a sister to *AGL6* (Kofuji et al., 2003; Nam et al., 2003; Parenicova, 2003; Zahn et al., 2005; Litt, 2007; *Amborella* Genome Project, 2013; Kim et al., 2013; Ruelens et al., 2013; Ubi et al., 2013; Wong et al., 2013; Yockteng et al., 2013). Interestingly, if the former scenario is correct, then it implies that both *AP1*/*FUL* and *SEP* have originated before the diversification of angiosperms; otherwise, it implies that both *AP1*/*FUL* and *SEP* have existed in the MRCA of extant seed plants but have been independently lost in the lineage leading to extant gymnosperms. The observation that the *FLOWERING LOCUS C* (*FLC*) may be the real sister of *AP1*/*FUL* (Ruelens et al., 2013) further complicated the issue, making it necessary to re-investigate the relationships among the aforementioned gene subfamilies.

Many factors, such as biased sampling, long-branch attraction, and heterogenous substitution rates, can lead to skewed topology of a phylogenetic tree (Kong et al., 2004; Leebens-Mack et al., 2005). However, the most important factor is the reliability of the alignment used for phylogeny estimation. Since using only conserved regions would reduce resolution, most studies include as many as possible alignable sites. Yet, it has recently been revealed that changes in the exon-intron structure of genes (i.e., structural changes, which may be caused by exon/intron gain/loss, exonization/pseudoexonization, and intraexonic insertion/deletion; Roy and Gilbert, 2005; Xu et al., 2012; Long et al., 2013) may hamper the homology-based alignment because they may lead to the addition of nonhomologous sequence or removal of homologous nucleotide. Since almost all studies only used coding sequences (CDS) or protein sequences to generate their alignment, nonhomologous sites caused by structural changes could be forced to align together. In the MADS-box gene family, structural changes have been shown to be rather common and can indeed cause shifts of reading frame (Litt and Irish, 2003; Vandenbussche et al., 2003a; Litt, 2007; Shan et al., 2007; Xu and Kong, 2007; Liu et al., 2011; Xu et al., 2012). A good example comes from comparing the three core eudicots lineages of the *AP1*/*FUL* subfamily: eu*FUL, AGL79* (also called core eudicot *FUL*-like), and eu*AP1* (Litt and Irish, 2003; Litt, 2007; Shan et al., 2007). Proteins encoded by the first two lineages have a paleoAP1 motif at the C-terminal region, the first six amino acids of which were also defined as FUL-like motif in some studies (Litt and Irish, 2003; Litt, 2007) and show high similarity with part of AGL6 II and SEP II motifs. The eu*AP1* lineage, however, encodes for a quite different C-terminal region with two different motifs: a transcription activation domain and a euAP1 motif, the final four amino acids of which were also called farnesylation motif (Litt and Irish, 2003; Litt, 2007). Detailed investigation revealed that the novel sequence was generated by a 1-bp deletion in exon 8 of the ancestral eu*AP1* gene (Litt and Irish, 2003; Vandenbussche et al., 2003a; Litt, 2007; Shan et al., 2007). Similarly, an 8-bp insertion (Vandenbussche et al., 2003a) or a 1-bp deletion (Kramer et al., 2006) in the last exon has likely given rise to a new eu*AP3* motif in the eu*AP3* lineage of the *AP3* subfamily. During phylogenetic reconstruction of the *AP1*/*FUL, SEP, AGL6*, and *FLC* subfamilies, however, none of the previous studies considered exon-intron structural changes when generating the final alignment, which may explain why different studies have obtained slightly different topologies.

In this article, we first investigated structural changes during the evolution of these and related subfamilies such as *SUPPRESSOR OF OVEREXPRESSION OF CO 1* (*SOC1*), *AG*, and *STK*. We found that structural changes have occurred frequently in these subfamilies and could indeed affect phylogenetic estimation and the understanding of gene evolution. With the knowledge of structural changes, we generated more reasonable alignments containing all the focal subfamilies. All the resulting trees support the sisterhood of *AP1*/*FUL* and *FLC*, with *SEP* and *AGL6* being successive sisters to them. In the context of this new topology, we discussed the contribution of structural changes to the origin and functional diversification of different subfamilies.

## Materials and methods

### Sequence retrieval and classification

The protein, coding, and genomic (if available) sequences of focal MADS-box genes were retrieved by BLAST searches against the GenBank (http://www.ncbi.nlm.nih.gov), FGP (http://fgp.bio.psu.edu), Phytozome (http://phytozome.jgi.doe.gov), *Amborella* Genome Database (http://www.amborella.org), TAIR (https://www.arabidopsis.org), MPOB (http://genomsawit.mpob.gov.my), and PlantGDB (http://www.plantgdb.org) databases, with multiple sequences being used as queries. The resulting dataset was then trimmed by the following strategies. First, CDSs shorter than 400 bp were excluded, because they are not very informative or accurate. Second, all but one of the multiple highly similar (i.e., >95% identical at the CDS level) sequences from the same species were eliminated, because they represent alleles of the same gene. Third, for genes with alternative splicing, only the transcript showing the least structural divergence from closely related homologs was adopted. And fourth, poorly annotated sequences from whole-genome sequenced species were excluded. As a result, 792 sequences were retained for further analyses.

To assign the retained sequences into different subfamilies, we built a preliminary phylogenetic tree (using the same methods described below) with shared regions (Dataset S1). The matrix for every subfamily has a broad taxonomic coverage, including sequences from early-diverging angiosperms, monocots, magnoliids, basal eudicots, core eudicots, and gymnosperm species (if applicable). Detailed information of genes included in this study was listed in Table S1.

### Sequence alignment and phylogenetic reconstruction

For each subfamily, protein sequences were initially aligned using ClustalX 1.83 with default options (Thompson et al., 1997), and its corresponding codon-based CDS alignment was generated by the PAL2NAL program (http://www.bork.embl.de/pal2nal/). A preliminary tree was constructed with the CDS alignment excluding poorly aligned regions (i.e., columns). The sequences in both protein and CDS alignments were then reordered according to their phylogenetic placements as well as the phylogenetic relationships among species. By comparing closely related sequences, we were able to determine homologous sites and refine the alignments. Considering the effect of structural changes on the reliability of alignment, we marked the exon-intron boundaries for genes with structural annotation (from genome-sequenced species) and carefully checked the alignments of neighboring sequences exon by exon. Special attention was paid to the exons that showed considerable divergence in sequences or lengths, in which structural changes have likely occurred. To improve the alignment quality, a pairwise alignment was performed by using both focal exons and their flanking noncoding sequences. Referring to these results, the CDS alignment can be adjusted with confidence, which were carried out in MEGA 6.0 (Tamura et al., 2013). Since our alignments involved human judgment and might be arbitrary, we also generated an amino acid alignment using Probalign (Roshan and Livesay, 2006) for each subfamily and its corresponding codon-based CDS alignment. Eventually, the CDS alignments excluding nonhomologous and highly divergent regions/sites were used for phylogenetic analyses.

To estimate the phylogenetic relationships among different subfamilies, we generated a combined matrix using the “profile-profile alignment” method in Muscle 3.6 (Edgar, 2004), followed by manual adjustments as described above. To maximize the reliability of our phylogenetic analyses, we created three different alignments (I, II, and III). For alignment I, all the 792 sequences were included. Alignment II contained 498 sequences with the exclusion of genes or gene lineages that experienced structural changes shortly after gene duplications. More stringently, in alignment III, we only included 57 exemplars from basal angiosperms, basal eudicots and gymnosperms (if applicable), which showed less structural divergence during evolution (for details, see results). Because no *FLC*-like gene has ever been identified from basal angiosperms and basal eudicots (Ruelens et al., 2013; this study), *FLC*-like genes from core eudicot species and *Musa* were used for this subfamily. For all the alignments, only homologous sites and regions were used for phylogenetic analyses (Dataset S9).

Phylogenetic relationships of genes within each subfamily were revealed by the maximum-likelihood (ML) method, which was performed on the DNA matrix with PhyML (version 2.4) (Guindon and Gascuel, 2003). The most appropriate molecular evolution model (GTR+I+Γ) was selected, following the estimate with MODELTEST version 3.06 (Posada and Crandall, 1998). A BIONJ tree was used as a starting point for ML searches (Guindon and Gascuel, 2003), and bootstrap analyses were performed with 100 replicates. In addition to the ML method, we also performed Bayesian inference (BI; Ronquist et al., 2012) for alignments I, II, and III to confirm the phylogenetic relationships among the *AP1*/*FUL, SEP, AGL6*, and *FLC* subfamilies. We ran four chains, sampling one tree every 1000 generations for 15,000,000 generations using GTR+I model (starting with a random tree). The first 25% trees were considered burn-in and discarded from further analysis.

### Determination of exon-intron structural changes

To understand the history of structural changes, we first determined the causal of each gap in the alignment and then tried to trace the origin of each gap on the phylogenetic tree. Gaps located at one or both sides of an exon could be caused by exonization/pseudoexonization or exon gain/loss events. The former could be inferred when exonic sequence of one gene was alignable with intronic or intergenic sequence of the other gene. The latter is the phenomenon when an entire exon of one gene could not be aligned to any region (including noncoding sequences) of the other. Gaps within an exon are usually caused by intraexonic insertions/deletions. We mapped the occurrence and the causal of each gap on the phylogenetic tree and deduced at which branch they have happened according to the maximum parsimony principle. In addition to the above mechanisms, intron gain/loss is also responsible for structural changes as previously reported (Xu et al., 2012), which was regarded when one exon of a certain gene could be perfectly aligned with two neighboring exons of the other gene. Different from other mechanisms, no gaps could be found in the alignment if intron gain/loss has happened, but it could lead to the difference in exon numbers. Therefore, the evolutionary history of intron gain/loss was also inferred. With the knowledge of these exon-intron structural changes, we estimated the exon-intron structures of the various ancestral genes in the MRCAs of extant core eudicots, Ranunculales, magnoliids, monocots, angiosperms, and gymnosperms (if applicable).

## Results

### Structural changes within the *AP1*/*FUL* subfamily

A total of 209 genes were used for the structural analysis of *AP1*/*FUL* subfamily members. By performing exon-by-exon alignment, we generated a dataset consisting of 711 nucleotide sites, among which 607 were phylogenetically informative (Dataset S1). The topology of the final phylogenetic tree was largely consistent with previous studies and not sensitive to missing data (Litt and Irish, 2003; Preston and Kellogg, 2006; Shan et al., 2007; Xu and Kong, 2007; Litt and Kramer, 2010; Pabón-Mora et al., 2013). Nonetheless, the resolution was slightly improved and the positions of most genes were better congruent with angiosperm phylogeny. In contrast, the dataset created based on an alignment produced by Probalign only included 696 sites, among which 591 were informative (Dataset S2). Moreover, in the resulting phylogenetic tree, the positions of some major plant groups were discordant with angiosperm phylogeny (Dataset S2). Similar results were obtained when other MADS-box gene subfamilies were analyzed (Datasets S3–S8). This suggests that phylogenetic estimation can indeed be improved when structural changes were taken into consideration during alignment.

**Figure 1:**
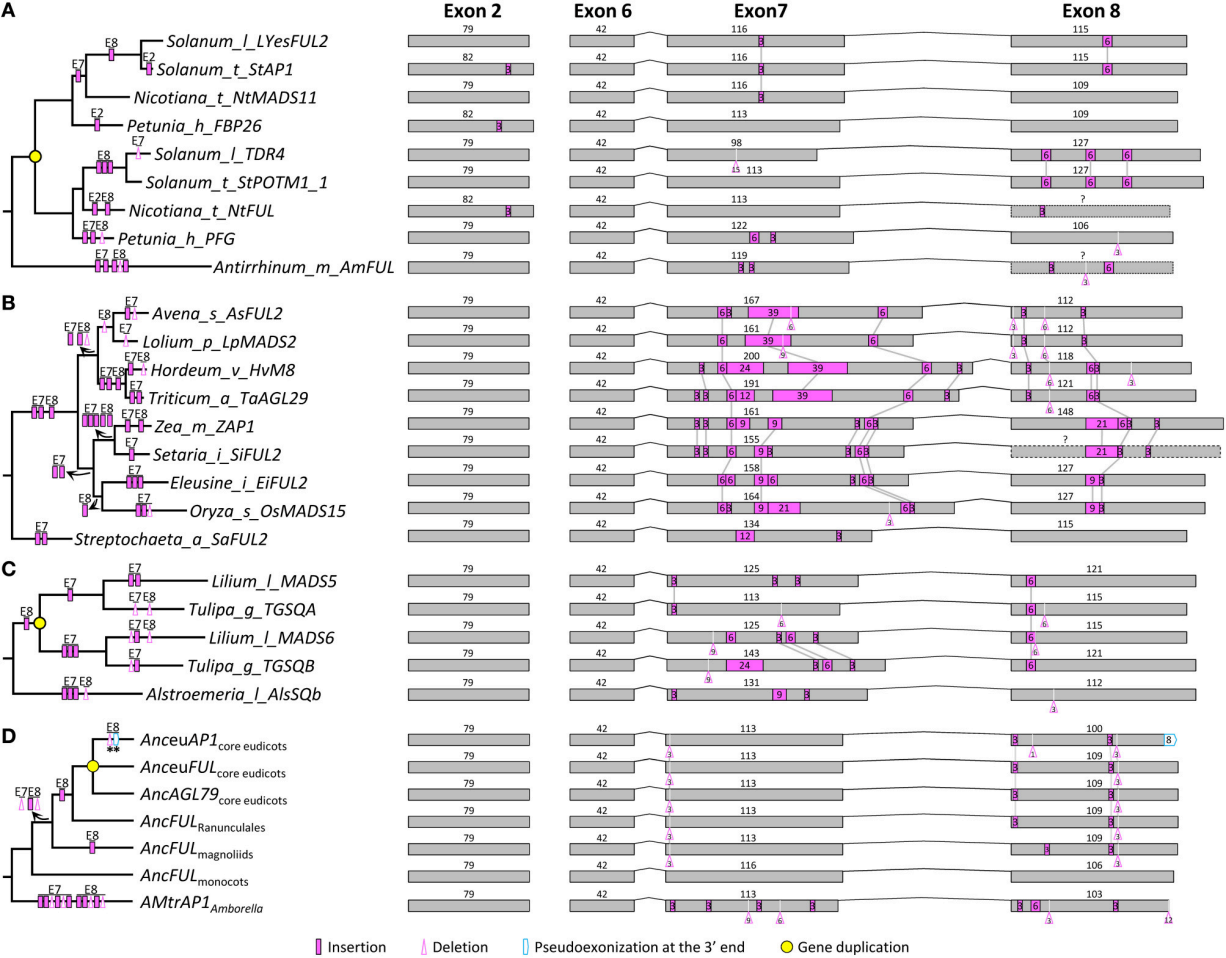
**Evolution of exon-intron structure in the *AP1*/*FUL* subfamily**. **(A–C)** Representative structural change events occurred in the eu*FUL* lineage of Solanaceae **(A)**, the *OsMADS15* lineage of Poaceae **(B)**, and *AP1*/*FUL*-like genes of Liliales **(C)**. **(D)** Exon-intron structural changes at several key nodes on the *AP1*/*FUL* phylogenetic tree. “Anc” (for Ancestor) is prefixed to the name of each gene lineage. Details are shown in Figure S1. Exons and introns are represented by boxes and curved lines, respectively. The length of each exon is shown above the box. Shared structural change events are linked by gray lines. Different mechanisms responsible for structural changes are marked on corresponding branches of the phylogenetic tree. Stars indicate structural changes involving non-triplet sequences.

In the context of the improved phylogeny, we attempted to trace the evolutionary changes in the exon-intron structure of *AP1*/*FUL* subfamily members. We found that the *AP1*/*FUL*-like genes generally consist of eight exons, among which the first six have been highly conserved. In contrast, exons 7 and 8 vary greatly in length (from 77 to 209 bp for exon 7 and 34 to 148 bp for exon 8), suggestive of dramatic structural changes (Figure S1). Detailed comparisons revealed that intraexonic insertion/deletion occurred more frequently than exonization/psedoexonization in this subfamily, and that structural changes were not distributed evenly among branches. For example, an average of 2 insertion/deletion events was detected in the Solanaceae eu*FUL*-like genes (Figure 1A), while at least 8 structural change events were observed for each of the *OsMADS15* lineage members (Figure 1B).

We also found many structural change events shared by certain plant groups or major gene lineages. For example, in exon 7, a 3-bp deletion was detected in all *OsMADS14*/*15* members of monocots, and two independent 3-bp insertions were found in the *OsMADS14* and *OsMADS15* lineages of Poaceae (Figure S1). In exon 8, one 3-bp insertion near the 5′ boundary was shared by all the sampled eudicot members (Figure S1), suggestive of an ancient structural change event occurred before the diversification of eudicots. There are also multiple cases where structural changes have caused divergence of duplicate genes. For instance, in the *OsMADS18*/*20* lineage of monocots, a gene duplication event resulted in the creation of two sublineages in Liliaceae (Figure 1C). The ancestor of one sublineage has experienced a 3-bp insertion in exon 7, while that of the other sublineage has undergone three insertions of different lengths in the same exon. Consistent with previous studies (Litt and Irish, 2003; Vandenbussche et al., 2003a; Shan et al., 2007), we also detected a 1-bp deletion in exon 8 of all examined eu*AP1*-like genes, which led to pseudoexonization of the last 8 nucleotides (Figure 1D). With the knowledge of these structural changes, we inferred that the *AP1/FUL*-like gene in the MRCA of extant angiosperms is composed of eight exons with the lengths of 185, 79, 65, 100, 42, 42, 113, and 106 bp, respectively.

### Structural changes within the *SEP* subfamily

**Figure 2:**
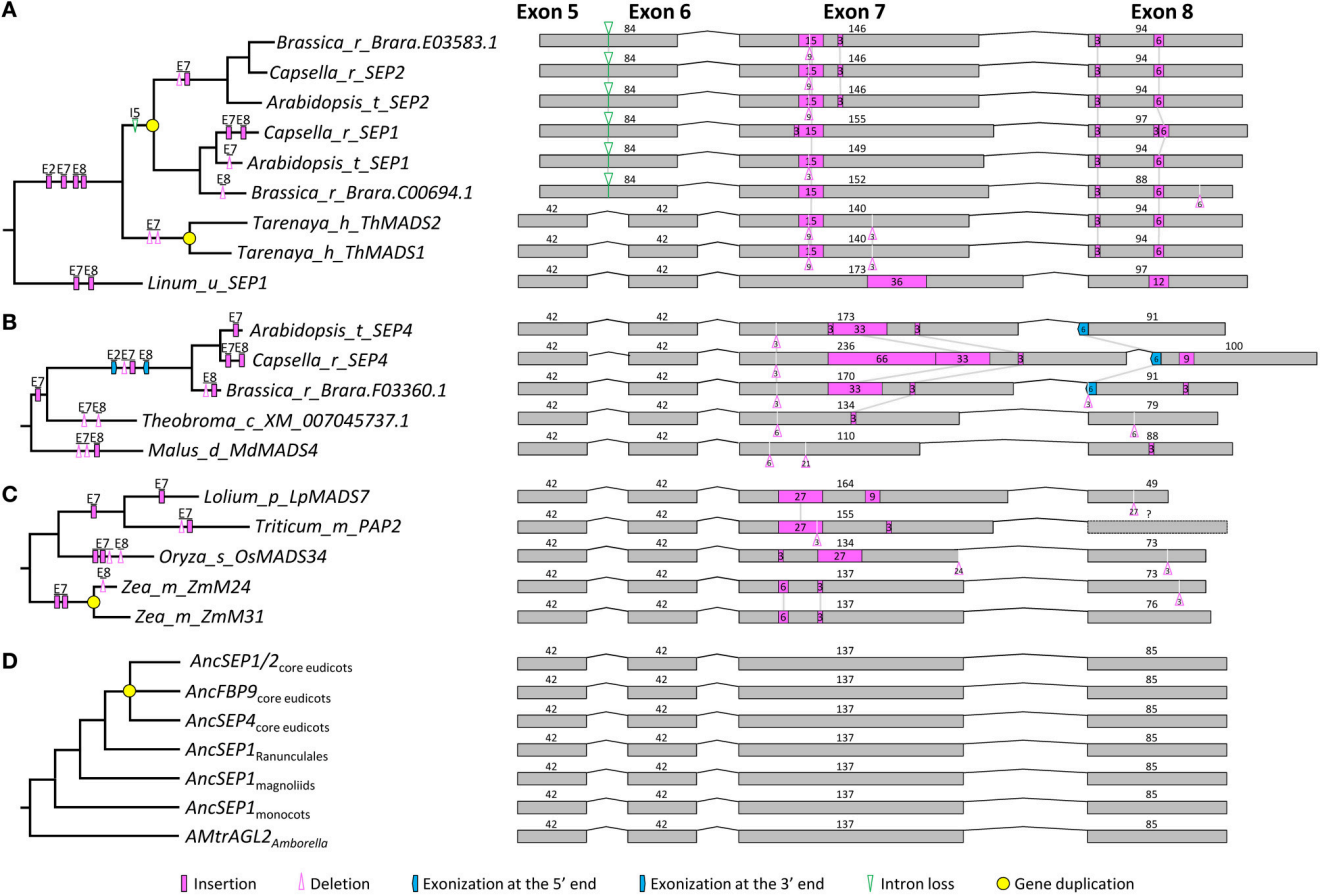
**Evolution of exon-intron structure in the *SEP1* subfamily. (A–C)** Representative structural change events occurred in *SEP1/2* **(A)** and *SEP4* **(B)** lineages of Brassicaceae, and the *OsMADS34* lineage of Poaceae **(C). (D)** Exon-intron structural changes at several key nodes on the *SEP1* phylogenetic tree. Details are shown in Figure S2. The symbols describing structural changes are the same as those in Figure 1.

We obtained 119 *SEP1*- and 87 *SEP3*-like genes to analyze exon-intron structural changes in the *SEP* subfamily. According to a previous study (Zahn et al., 2005) and this study, *SEP1*-like genes contain three major lineages in both core eudicots (i.e., *SEP1*/*2, FBP9*, and *SEP4*) and grasses (i.e., *OsMADS1, OsMADS5*, and *OsMADS34*; Figure S2). Except for *SEP1/2*-like genes in Brassicaceae and *EgAGL2-5* in *Elaeis guineensis*, all these genes have eight exons. For the Brassicaceae *SEP1*/*2*-like genes, the fifth exon (84 bp) could be aligned perfectly to the fifth (42 bp) plus the sixth (42 bp) exon of other genes, suggestive of an intron loss event that occurred before the diversification of Brassicaceae (Figure 2A). Like the situation in the *AP1*/*FUL* subfamily, structural change events were mostly observed in the seventh and eighth exons, but the occurrence frequency was much lower (Figure 2 and Figure S2). A large number of structural changes could be found before the diversification of certain plant groups. For example, one 3-bp insertion in exon 2, one 15-bp insertion in exon 7, and two insertions (3 and 6 bp, respectively) in exon 8 of *SEP1/2*-like genes have likely occurred in the MRCA of Brassicaceae and Cleomaceae (Figure 2A and Figure S2). The longest insertion (66 bp) was observed in exon 7 of the *SEP4* gene of *Capsella rubella*, adjacent to which was an extra 33-bp insertion that has occurred in the ancestor of this and two other related species (*Brassica rapa* and *Arabidopsis*; Figure 2B). There were also evidences showing the contribution of structural changes to the divergence of duplicate genes. For instance, maize has a pair of duplicate genes (*ZmM24* and *ZmM31*) in the *OsMADS34* lineage. A 3-bp deletion happened in exon 8 of *ZmM24*, making the lengths of this exon different between them (Figure 2C). In addition to recent duplicates, structural changes in more ancient duplicates were also detected. One 3-bp deletion event in exon 2 of the *OsMADS1* lineage, as well as one 45-bp pseudoexonization event in exon 8 of the *OsMADS5* lineage, has likely taken place before the diversification of grasses (Figure S2). Within the *SEP1* clade, no structural change event has likely occurred before the origins of major plant groups (i.e., monocots, magnoliids, and core eudicots; Figure 2D and Figure S2). Based on this information, we inferred that the *SEP1*-like gene in the MRCA of extant angiosperms contains eight exons, with the lengths of 185, 79, 62, 100, 42, 42, 137, and 85 bp, respectively.

**Figure 3:**
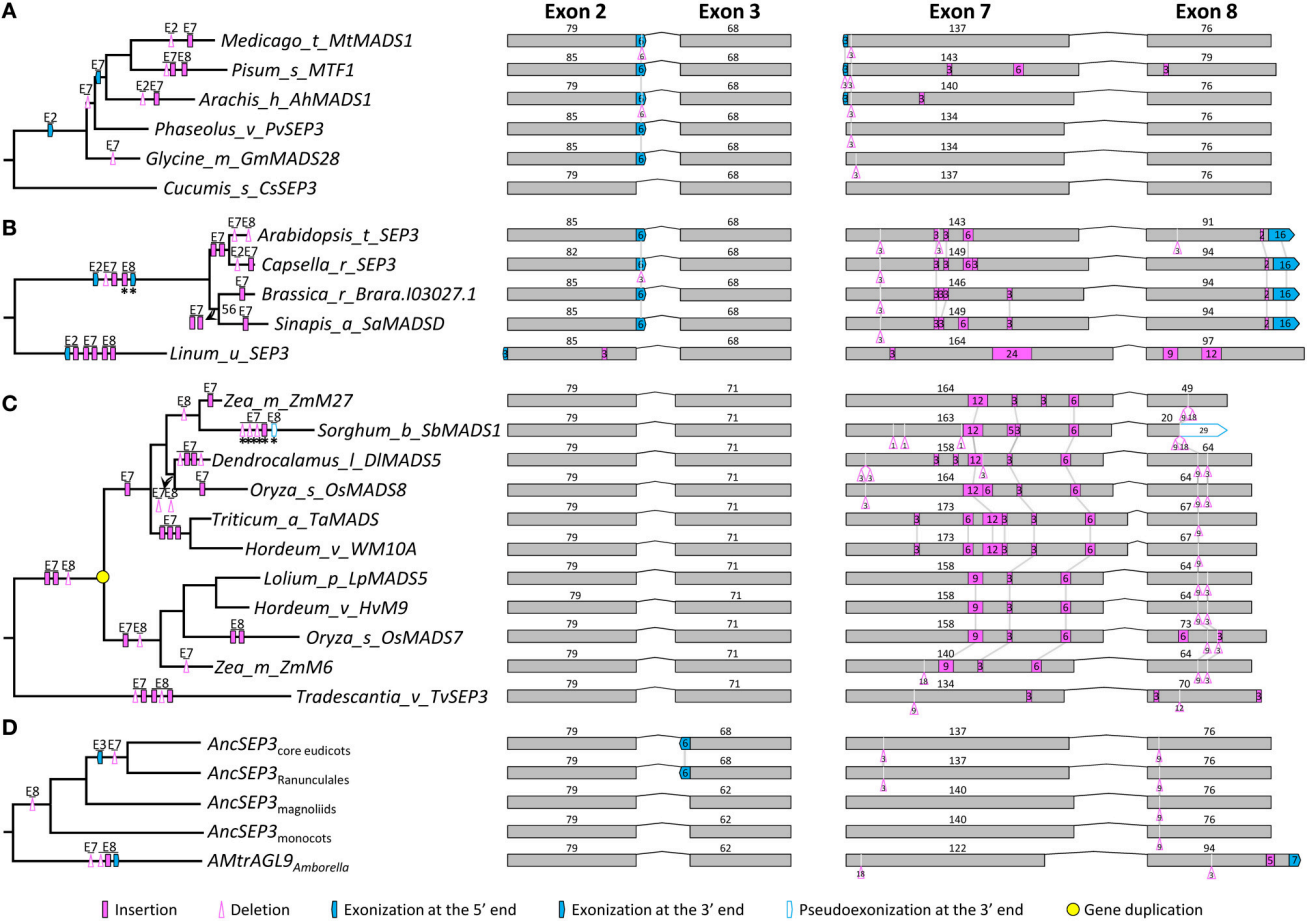
**Evolution of exon-intron structure in the *SEP3* subfamily. (A–C)** Representative structural change events occurred in *SEP3*-like genes of Fabaceae **(A)**, Brassicaceae **(B)**, and Poaceae **(C). (D)** Exon-intron structural changes at several key nodes on the *SEP3* phylogenetic tree. Details are shown in Figure S3. The symbols describing structural changes are the same as those in Figure 1.

The phylogenetic tree of *SEP3*-like genes indicates no major gene duplication event (Figure S3). All of the 87 genes have eight exons. For exons 1, 4, 5, and 6, the lengths are largely conserved (185, 100, 42, and 42 bp, respectively) with a few exceptions (Figure S3). Exons 2, 3, 7, and 8, in contrast, vary remarkably in length, suggestive of multiple structural changes (Figure 3 and Figure S3). For exon 2, independent exonization events were observed in several taxa, such as Fabaceae, Brassicaceae, and *Eupomatia*, among others (Figures 3A,B, and Figure S3). In exon 3, a 9-bp exonization event was detected in members of Asparagales, Commelianales, and Poales, suggestive of an early structural change event during the evolution of monocots. Still in this exon, a more ancient exonization (6 bp) event was found before the divergence of Chloranthaceae (Figure S3). In exon 7, the MRCA of eudicots has experienced a 3-bp deletion event, while that of grasses has undergone two independent insertion events (Figures 3B,C and Figure S3). The earliest structural change event was a 9-bp deletion in exon 8, which happened after the divergence of *Amborella trichopoda* (hereafter called *Amborella*; Figure 3D). Taking into account of all the structural change events, we estimated that the *SEP3*-like gene in the MRCA of extant angiosperms contains eight exons, the lengths of which are 185, 79, 62, 100, 42, 42, 140, and 85 bp, respectively.

### Structural changes in the *AGL6* subfamily

**Figure 4:**
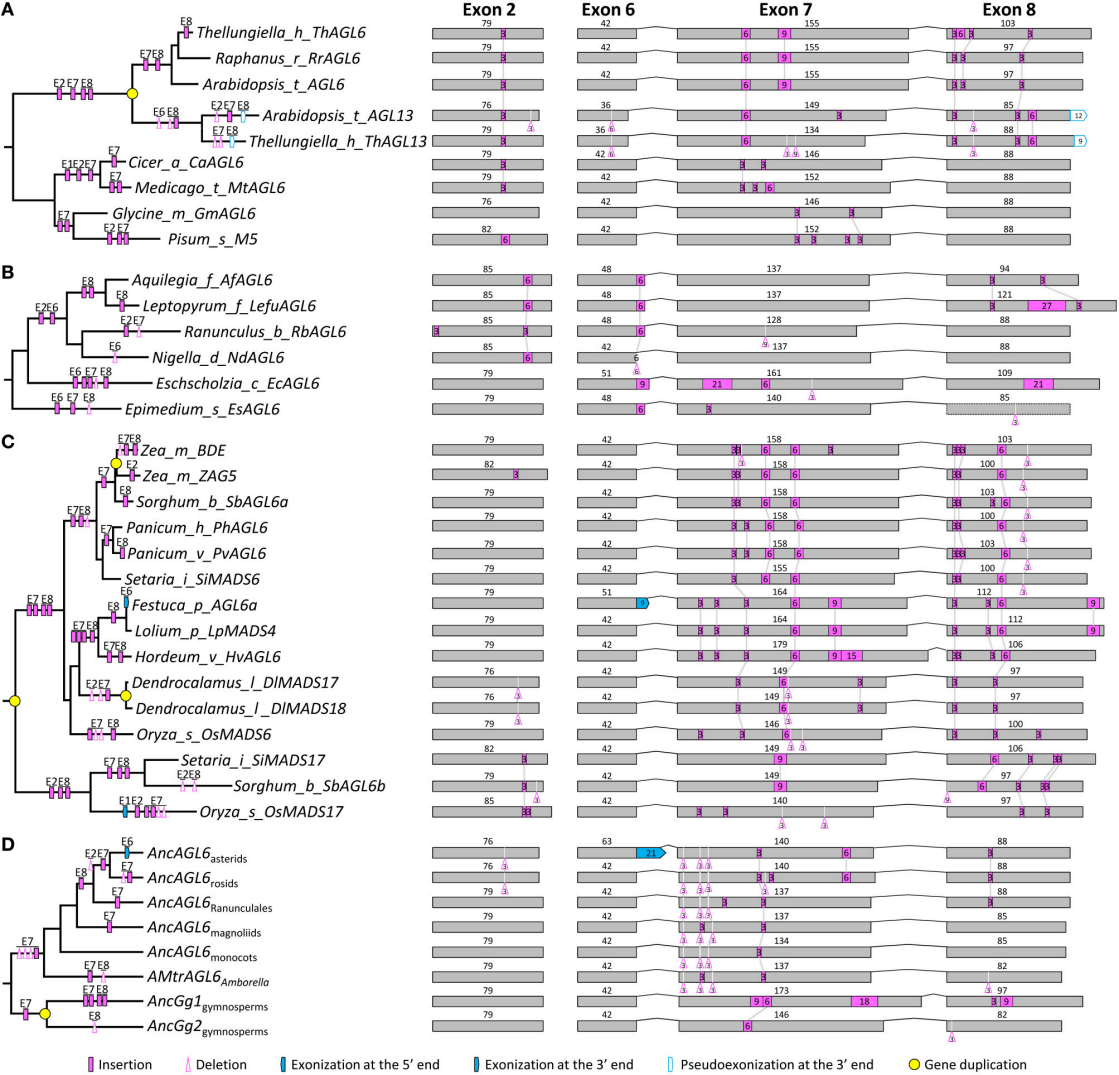
**Evolution of exon-intron structure in the *AGL6* subfamily. (A–C)** Representative structural change events occurred in *AGL6*-like genes of Brassicaceae **(A)**, Ranunculaceae **(B)**, and Poaceae **(C). (D)** Exon-intron structural changes at several key nodes on the *AGL6* phylogenetic tree. Details are shown in Figure S4. The symbols describing structural changes are the same as those in Figure 1.

Within the *AGL6* subfamily, 119 genes from angiosperms and 13 from gymnosperms were used for structural change analyses. The topology of the *AGL6* gene tree was similar to previous studies (Li et al., 2010; Kim et al., 2013). All the sampled genes except for *ZfAGL6a* in *Zamia fischeri* possess eight exons (Figure S4). The lengths of exons 1, 3, 4, and 5 (182, 62, 100, and 42 bp, respectively) are largely the same with exceptions in only five genes. In exon 2, other than a 3-bp deletion event occurred before the diversification of core eudicots, multiple independent insertion events were detected in several taxa, such as Brassicaceae and Ranunculaceae (Figures 4A,B, and Figure S4). In exon 6, a 21-bp exonization event occurred in the MRCA of asterids (Figure S4). Like the situation in the above two subfamilies, exons 7 and 8 were subject to multiple structural change events. In exon 7, major events include a 6-bp insertion in the MRCA of extant gymnosperms, a 3-bp insertion and three independent 3-bp deletions in the MRCA of extant angiosperms, a 3-bp insertion in the MRCA of Ranunculales, a 6-bp insertion in the MRCA of core eudicots, a 3-bp insertion and a 3-bp deletion in the MRCA of rosids, a 3-bp insertion and a 3-bp deletion in the MRCA of Asteraceae, a 6-bp insertion in the MRCA of Brassicaceae, and two 3-bp insertions in the MRCA of Poaceae (Figures 4A,D and Figure S4). In exon 8, independent insertion/deletion events were observed prior to the origins of eudicots, Asteraceae, and Poaceae, respectively (Figure S4). Structural divergence after gene duplication was also not a rare case in this subfamily. For example, *OsMADS6* and *OsMADS17* are two lineages generated by the pre-Poaceae gene duplication event, subsequent to which the former lineage went through two insertions in each of exon 7 and exon 8, while the latter experienced a 3-bp insertion in exon 2 and two 3-bp insertions in exon 8 (Figure 4C). Independent insertion/deletion events were also found in the duplicate lineages (Gg1 and Gg2) of gymnosperms (Figure 4D and Figure S4; Li et al., 2010). Considering all these structural change events, we inferred that the *AGL6*-like gene in the MRCA of extant angiosperms contains eight exons, with the lengths of 182, 79, 62, 100, 42, 42, 134, and 85 bp, respectively.

### Structural changes within the *FLC* subfamily

A recent study showed that *FLC*-like genes form a sister group to the *AP1*/*FUL* subfamily, and are closely related to the *SEP* and *AGL6* subfamilies (Ruelens et al., 2013). By carefully examining the sequences and deeply mining all available plant genomic data, we found that, as Ruelens et al. (2013) revealed, *FLC*-like genes could only be identified in core eudicots, Poaceae, and *Musa* (Musaceae). These findings suggest that *FLC*-like genes may have been lost independently in several lineages of angiosperms (Ruelens et al., 2013). Our phylogenetic tree showed that the *FLC*-like genes form two clades. One clade contains genes from core eudicots, including *FLC* and *MAF1/2/3/4/5* lineages generated by a pre-Brassicaceae gene duplication event; the other is composed of monocot genes, including *OsMADS51* and *OsMADS37* lineages produced by a pre-Poaceae gene duplication event. Unlike the aforementioned subfamilies, the core eudicot *FLC*-like genes have seven exons and exons 1, 4, 5, and 6 (185, 100, 42, and 42 bp, respectively) are evolutionarily conserved. In contrast, most monocot genes possess only five exons (Figure S5). Given the dramatic divergence of exon-intron structures of the *OsMADS37*-lineage genes, they were excluded from further analysis.

In the context of the phylogeny, we traced the history of structural changes in this subfamily. We found that some structural change events were shared by core eudicot genes or Brassicaceae genes. In Poaceae, multiple structural change events are likely to have happened in the ancestor of the *OsMADS51* lineage. For example, an intron loss event was detected in exon 5 because it could be aligned to the fifth and sixth exons of core eudicot genes. The last exon, which is the counterpart of the seventh exon in genes from core eudicots, probably has been lost; however, due to rapid sequence evolution of this subfamily, the underlying mechanism is hard to determine. Other relatively trivial structural change events include a 3-bp insertion and a 3-bp deletion in exon 1, a 3-bp insertion and a 15-bp deletion in exon 3, and a 3-bp deletion in exon 4 (Figure S5). Based on these analyses, we inferred that the *FLC*-like gene in the MRCA of extant angiosperms has lost an exon and thus contains seven exons, with the lengths of 185, 79, 68, 100, 42, 42, and 105 bp, respectively.

### Structural changes within the *SOC1, AG*, and *STK* subfamilies

Structural changes of the outgroup subfamilies (*SOC1, AG*, and *STK* subfamilies) were also examined, which show relatively close relationships with the *AP1*/*FUL, SEP, AGL6*, and *FLC* subfamilies (Kim et al., 2005, 2013; *Amborella* Genome Project, 2013; Ruelens et al., 2013). *SOC1* subfamily members are present in both angiosperms and gymnosperms. All genes from monocots form a monophyletic clade with moderate bootstrap support (72%), with Poaceae genes falling into three lineages (*WSOC1, TaAGL7*, and *TaAGL23*). Within core eudicots, another three lineages, each containing genes from rosids and asterids, may have been generated by the γ genome triplication event (Tang et al., 2008). Here we named them eu*SOC1, AGL42/71/72*, and *AGL14/19* after the homologs in *Arabidopsis* (Figure S6). All except for three *SOC1*-like genes (i.e., Brara.I00679.1 in *Brassica rapa, SOC1* in *Linum usitatissimus*, and *CsSOC1B* in *Cucumis sativus*) are composed of seven exons. For the first six exons, only a few structural change events were detected, which sparsely distributed across the angiosperm clade. Most structural changes were found in exon 7, including multiple insertion/deletion and exonization/psuedoexonization events (Figure S6). Taken together, we inferred that the *SOC1*-like gene in the MRCA of extant seed plants likely contains seven exons, with the lengths of 182, 82, 62, 100, 42, 42, and 132 bp, respectively.

The phylogenetic relationships of the *AG* and *STK* subfamilies were largely consistent with a previous study (Zahn et al., 2006), with the majority of genes containing seven exons (Figure S7). Structural analyses revealed several major structural changes in the *AG* subfamily, such as a 3-bp insertion in exon 7 after the divergence of *Amborella* and a 6-bp insertion in exon 7 before the diversification of eudicots. In the *STK* subfamily, one 3-bp exonization event in exon 3 and two separate insertions in exon 7 have occurred in the MRCA of monocots (Figure S7). Tracing back to the MRCA of extant seed plants, we concluded that the ancestral *AG/STK*-like gene contains seven exons, with the lengths of 182, 82, 62, 100, 42, 42, and 159 bp, respectively.

### Phylogenetic relationships and structural differences among subfamilies

**Figure 5:**
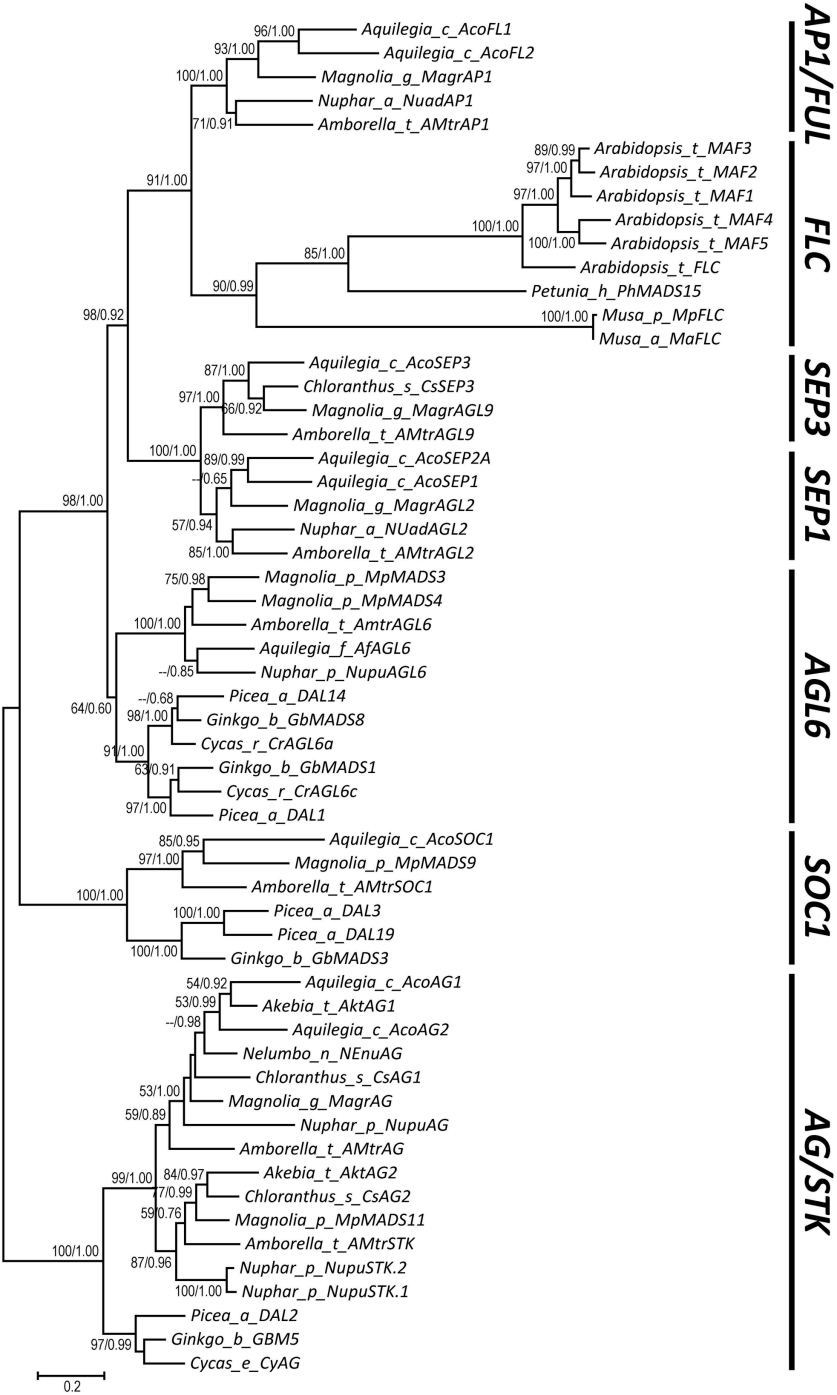
**A phylogenetic tree showing relationships of the *AP1/FUL*, *FLC*, *SEP*, and *AGL6* subfamilies**. The bootstrap values (>50%) obtained from maximum likelihood analysis and the posterior probabilities (>0.5) estimated by Bayesian inference are shown next to the nodes.

To resolve the relationships among all focal subfamilies, we constructed phylogenetic trees with three different matrices (alignments I, II, and III) (see Section Materials and Methods; Dataset S9). Topologies of all three trees were largely consistent, but the nodal supports at key nodes increased as more structurally diverged sequences were removed (Figure S8 and Figure 5). In the first tree, which was constructed using the matrix composed of all 792 sequences (alignment I), *AP1*/*FUL* and *FLC* are sisters, with 57% ML bootstrap support (BP) and 0.99 Bayesian posterior probabilities (PP), and *SEP* is the sister to them (50% BP and 0.97 PP). *AGL6* shows a sister relationship with the abovementioned three subfamilies (89% BP and 1.00 PP; Figure S8A). Considering that duplicate genes usually show accelerated evolutionary rate and more frequent structural changes that may screw the phylogeny, we next removed duplicated genes that diverged greatly in structure and generated a second matrix (alignment II). The tree built using this matrix gained increased supports for almost all of the abovementioned nodes (Figure S8B). To further improve the resolution, we selected genes (alignment III) with more conserved exon-intron structure from the second matrix and constructed the third tree. All focal nodes were strongly supported in both ML and BI trees (Figure 5).

**Figure 6:**
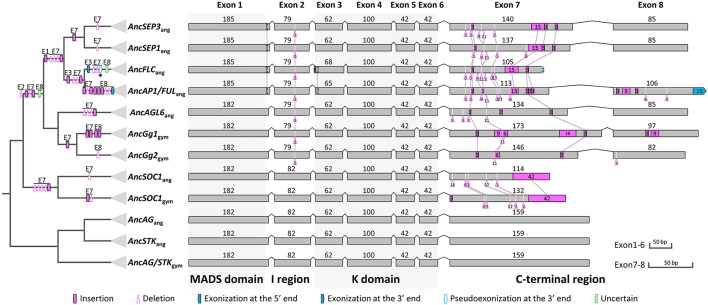
**Evolution of exon-intron structures of the *AP1*/*FUL*, *FLC*, *SEP*, and *AGL6* subfamilies**. The simplified tree is from Figure 5 and Figure S8. Show here is the ancestral exon-intron structure of each subfamily in the MRCA of extant angiosperms and in the MRCA of extant gymnosperms (if applicable). The MADS domain, I region, K domain, and C-terminal region are indicated below exons, and the MADS and K domains are highlighted with gray boxes. “ang” is the abbreviation for “angiosperms,” and “gym” for “gymnosperms.” The symbols describing structural changes are the same as those in Figure 1.

Based on our alignment and the topology of the resultant phylogenetic trees, we traced the evolutionary changes of exon-intron structures in these subfamilies. As described earlier, in the MRCA of extant angiosperms or seed plants (if applicable), the *AP1*/*FUL, SEP*, and *AGL6* genes all possess eight exons, while the *FLC, AG/STK*, and *SOC1* genes all contain seven exons (Figure 6). Unambiguous homologous relationships of exon 1 to exon 6 could be determined based on conservation of the encoded amino acid sequences, i.e., the MADS domain, I region, and K domain. Structural change events were found in exons 1, 2, 3, 7, and 8, some of which were shared by different subfamilies and consistent with their phylogenetic relationships (Figure 6). In exon 1, Kim et al. (2013) found a 3-bp gap in all *AGL6*-like genes but not in the *AP1*/*FUL* and *SEP* subfamilies. Here we found that this gap also appears in genes of *AG/STK* and *SOC1* subfamilies, suggesting a 3-bp insertion in the ancestor of *AP1*/*FUL, FLC*, and *SEP* subfamily genes (Figure 6). In exon 2, a 3-bp deletion has likely occurred in the ancestor of *AP1*/*FUL, FLC, SEP*, and *AGL6* subfamily genes. The length of exon 3 in all except for the *AP1*/*FUL* and *FLC* subfamilies is 62 bp. A 3-bp insertion plus an independent 3-bp exonization have resulted in an exon of 65 bp in the ancestor of the *AP1*/*FUL* subfamily and 68 bp in that of the *FLC* subfamily.

In all these subfamilies, exons 7 and 8 (if present), which encode(s) for the C-terminal region, is highly variable but contains short, relatively conserved, lineage-specific motifs. We found that in exon 7, the AG II motif (Kramer et al., 2004) was alignable to the SEP I motif (Zahn et al., 2005), the FUL motif (Shan et al., 2007), and the AGL6 I motif (Ohmori et al., 2009), and that the last four amino acids (LxxG) are quite conserved. This suggests that the seventh exons of different subfamilies (Figure S9) are homologous. In this exon, two 3-bp insertions and one 21-bp deletion have occurred before the divergence of *AP1*/*FUL, FLC, SEP*, and *AGL6* subfamilies. Three deletions with lengths of 3-, 3-, and 9-bp, respectively, as well as a 15-bp insertion were shared by the *AP1*/*FUL, FLC* and *SEP* subfamilies. The ancestor of *AP1*/*FUL* and *FLC* subfamily genes has likely experienced two deletion events. A 3-bp insertion shared by the *SEP* subfamily genes was also observed (Figure 6). These shared structural change events provide further support for the phylogenetic relationships among the four subfamilies.

Exon 8 is specific for the *AP1/FUL, SEP*, and *AGL6* subfamilies. Based on our phylogeny, it is highly likely that this exon originated before the divergence of these subfamilies. To figure out the mechanisms responsible for the evolutionary changes of this exon, we further searched putatively homologous sequences of this exon at the downstream 200 kb intergenic region of representative genes from the *FLC, SOC1, AG*, and *STK* subfamilies. However, due to the relatively long divergence time, we could not find any alignable region. Thus it is hard to determine whether this exon was generated by exonization or exon gain in the ancestor of the four subfamilies. Likewise, it is difficult to determine how this exon was lost in the *FLC*-like genes. More interestingly, we found that the ancestor of the *AP1*/*FUL* subfamily has experienced an exonization event at the 3′ boundary of exon 8. As we mentioned earlier, except for euAP1 proteins, all the other members of this subfamily encode for a paleoAP1 motif (Vandenbussche et al., 2003a; Shan et al., 2007), the first six amino acids of which is defined as FUL-like motif (Litt and Irish, 2003; Litt, 2007) and could be aligned to the C-terminal ends of the SEP and AGL6 proteins (Figure S10). To understand the origin of the extra 5 amino acids in the paleoAP1 motif, we tried to align the coding sequence of this region to the 3′ untranslated regions of *SEP* and *AGL6* subfamily genes. The resultant alignment (Figure S10) suggested that two point mutations (T–C and A–C) may have broken the original stop codon in the ancestor of the *AP1*/*FUL* subfamily, thereby leading to exonization of the next in-frame 15 bp and thus addition of new amino acids in the protein product (Figure S10). Intriguingly, the *Amborella* AMtrAP1 does not contain the extra 5 amino acids. Further investigation showed that this may have been caused by independent insertions and point mutations because the corresponding region in this species does not show much similarity with other *AP1*/*FUL*-like genes, or with *SEP* or *AGL6* subfamily members.

## Discussion

### Prevalence and functional impacts of exon-intron structural changes

Although previous studies have reported structural changes in MADS-box genes (Litt and Irish, 2003; Vandenbussche et al., 2003a; Kramer et al., 2006; Shan et al., 2007; Xu and Kong, 2007; Xu et al., 2012; Fourquin et al., 2013), it is ours that first trace the evolution of them in several subfamilies. By conducting such a detailed analysis, we found that: (1) structural changes are highly prevalent during the evolution of MADS-box genes, which contributed to the divergence of genes within and among subfamilies; (2) as has been shown in previous studies (Xu and Kong, 2007; Xu et al., 2009, 2012; Liu et al., 2011), structural changes could be achieved by three types of mechanisms, i.e., exon/intron gain/loss, exonization/pseudoexonizaiton, and intraexonic insertion/deletion; (3) although structural changes can occur in every exon, most of them took place in exons or the part of an exon that encodes for the I region or the C-terminal region; (4) most structural changes were fixed in a specific gene or species, but some important ones were preserved over long evolutionary time. Clearly, these results provide a comprehensive and updated insight into the significant role that structural changes have played in the diversification of gene families.

The frequent occurrence of structural changes in the C-terminal region is not surprising because it has long been demonstrated that this region varies considerably in length and sequence among MADS-box proteins. However, highly variable as it is, this region contains quite conserved motifs. Structural changes rarely occurred in these motifs, but when they did, they could occasionally cause the formation of new motifs (Litt and Irish, 2003; Vandenbussche et al., 2003a; Kramer et al., 2006; Litt, 2007; Shan et al., 2007). One typical example is the generation of the euAP3 motif by either insertion of eight nucleotides (Vandenbussche et al., 2003a) or deletion of one nucleotide (Vandenbussche et al., 2003a) in an ancestral paleoAP3-motif encoding gene. Another example is the generation of two new motifs in euAP1 proteins by 1-bp deletion (Litt and Irish, 2003; Vandenbussche et al., 2003a; Kramer et al., 2006; Litt, 2007; Shan et al., 2007). The above examples both involve out-of-frame insertions/deletions, which are generally deleterious. However, when occurring in duplicate genes, the presence of a redundant copy could compensate for the possible loss of function caused by frameshift mutations, enabling these mutations to lead to functional divergence (Raes and Van de Peer, 2005). As a previous study suggested, this might be the main pattern for novel motif generation in transcription factor families (Vandenbussche et al., 2003a). Interestingly, we found that other structural change mechanisms could also contribute to the generation of novel motifs. For example, the paleoAP1 motif was created by degeneration of the original stop codon and exonization of adjacent 15 nucleotides. More dramatically, the eighth exon, part of which encodes for conserved motifs in the *AP1/FUL, SEP*, and *AGL6* subfamilies, was likely generated by an exonization or exon gain event. These new motifs, which have been highly conserved for a remarkably long evolutionary time, are likely of extraordinary importance and could be a good starting point for functional studies.

Currently, there are only limited data on the functions of several C-terminal motifs and the results are conflicting. For example, one study showed that the euAP3 motif endowed euAP3-like proteins with new functions in specifying perianth structures in core eudicots (Lamb and Irish, 2003); whereas two other studies demonstrated that this motif was dispensable for floral organ identity determination (Piwarzyk et al., 2007; Su et al., 2008). The transactivation domain could indeed confer activation capability to euAP1-like proteins of *Arabidopsis*, radish (*Raphanus sativus*), and tobacco (*Nicotiana tabacum* and *Nicotiana sylvestris*; Cho et al., 1999). However, a couple of functional studies showed that euFUL and FUL-like proteins were able to substitute for AP1, indicating that the C-terminal motifs may not be essential for the functions of euAP1-like proteins (Gocal et al., 2001; Jang et al., 2002; Chen et al., 2008). Also, Krizek and Meyerowitz (1996) presented evidence that the C-terminal domains of AP1 and AG are not necessary for functional specificity. These opposing results may have been caused by different experimental methods, or possible redundancy of these proteins in high-order complexes (Litt and Kramer, 2010). Further investigations are needed in the future to address this question.

### Effects of structural changes on alignment and phylogenetic relationships among the *AP1*/*FUL, SEP, AGL6*, and *FLC* subfamilies

A reliable alignment is extremely important for the accuracy of phylogenetic estimation. Sequence similarity is empirically considered as a hint for homology; however, when evolutionary time is too long, it would be quite difficult to draw an unambiguous conclusion. In the present study, we demonstrated that structural changes are common during the evolution of a gene subfamily, and would directly or indirectly disrupt the homology of corresponding sites or regions in a couple of ways. First, insertion/deletion or exonization/pseudoexonization of non-triplet sequences would lead to shifts of reading frame and thus destroy homology of the downstream coding region. Second, independent changes at the same position in different species may be aligned together and thus erroneously produce nonhomologous sites in the matrix. We found quite a few such cases, one of which is several independent exonization events in exon 2 of core eudicot *SEP3*-like genes (Figure S3). Third, when a certain position is a hot spot for insertion/deletion, it would be hard to determine whether corresponding sites are homologous or not. This phenomenon has been observed frequently in grass genes (Figures S1, S3–S4, S6–S7). Finally, a structural change event may occur within a codon, and thus the homology is interrupted. Multiple cases have been found in this study, such as independent exonization events at the 5′ end of exon 8 in some genes of the *AP1*/*FUL* and *SEP* subfamilies (Figures S1–S3).Therefore, with the accessibility of more complete genome sequences, it is feasible to generate a more reasonable alignment by referring to exon-intron structure information.

In this study, with the knowledge of structural changes in each subfamily, we refined our alignments and estimated phylogenetic relationships of the *AP1*/*FUL, FLC, SEP*, and *AGL6* subfamilies. Our tree showed that *SEP* is sister to the monophyletic group formed by *AP1*/*FUL* and *FLC*, and that *AGL6* is the sister to the three abovementioned subfamilies. The topology is different from the one reported by Ruelens et al. (2013), in which *SEP* and *AGL6* are sister to each other and together they are nested with the lineage formed by *AP1*/*FUL* and *FLC*. Based on their phylogenetic tree and syntenic evidence, Ruelens et al. (2013) proposed that the ancestor of *AP1*/*FUL, FLC, SEP*, and *AGL6* subfamily genes experienced a tandem duplication event in the MRCA of extant seed plants, creating the ancestor of *SEP* and *AGL6*, and the ancestor of *AP1*/*FUL* and *FLC*. Then the former went through a duplication event and generated ancestral *SEP* and *AGL6* genes. The segment containing the ancestral *SEP* and the ancestor of *AP1*/*FUL* and *FLC* was then lost in the MRCA of extant gymnosperms. However, according to our phylogenetic tree and taking the syntenic evidence into account, we hypothesize that the ancestor of *AGL6, SEP, AP1*/*FUL*, and *FLC* has experienced a duplication event in the MRCA of extant seed plants, generating the ancestral *AGL6* and the ancestor of *SEP, AP1*/*FUL*, and *FLC*. The latter was then lost in the MRCA of extant gymnosperms but went through a tandem duplication event prior to the origin of angiosperms, bringing forth the ancestral *SEP* and the ancestor of *AP1*/*FUL* and *FLC*. Then the two genes underwent a whole genome duplication event in the MRCA of extant angiosperms and created *SEP1* and *SEP3*, and *AP1*/*FUL* and *FLC*, respectively. Our hypothesis is equally parsimonious with that of Ruelens et al. (2013) and the phylogenetic tree also showed stronger supports at key nodes than previous studies (Carlsbecker et al., 2003; Kim et al., 2005; Futamura et al., 2008; Li et al., 2010). Moreover, structural changes shared by different subfamilies provide extra evidence for our topology (Figure 6). The gradual improvement of nodal supports with successive removal of structurally diverged sequences suggests that structural changes could indeed influence sequence alignment and then phylogenetic estimation, which need to be carefully considered when studying the evolution of a certain gene family.

### Structural diversification is associated with functional divergence among subfamilies

Our results showed that structural changes have taken place in all the focal subfamilies but with different extents. The divergence pattern is significantly associated with their functions. For example, *SEP*-like genes have experienced much less structural changes than the *AP1/FUL, FLC*, and *AGL6* subfamily genes during evolution. Accumulating evidences have shown that the *SEP* subfamily members play conserved and vital roles in specifying floral organ identities of angiosperms. Silencing or mutation of *SEP*-like genes in different species, such as *Arabidopsis SEP1/2/3/4*, petunia *FBP2/FBP5*, tomato *TM5*/*TM29, Nigella damascena NdSEP1/2/3*, and rice *OsMADS1/5/7/8*, can lead to the transition of floral organs to sepal-, bract-, or leaf-like organs (Pnueli et al., 1994; Pelaz et al., 2001; Ampomah-Dwamena et al., 2002; Ferrario et al., 2003; Vandenbussche et al., 2003b; Ditta et al., 2004; Cui et al., 2010; Wang et al., 2015). Biochemical data revealed that the SEP-like proteins are able to form quaternary complexes with other floral MADS-box proteins in many species, such as *Arabidopsis*, petunia, *Gerbera hybrida, Vitis vinifera*, and rice (Honma and Goto, 2001; Ferrario et al., 2003; Ruokolainen et al., 2010; Seok et al., 2010; Smaczniak et al., 2012; Mellway and Lund, 2013). Recently, we reported that heterodimers between the SEP-like proteins and other floral MADS-box proteins can be formed in early diverging angiosperms, such as *Amborella* and *Nuphar pumila* (*Amborella* Genome Project, 2013; Li et al., 2015). Moreover, by conducting yeast two-hybrid assays with resurrected proteins of the MRCA of extant angiosperms, we found that the ancestral SEP-like proteins have broad interactions with other ancestral floral MADS-box proteins (Li et al., 2015). Therefore, it is highly likely that the *SEP*-like gene in the MRCA of extant angiosperms has obtained the function of determining floral organ identities and the ability to mediate the formation of floral quartets, which has been retained during the evolution due to their stable gene structures and conserved sequence features.

Unlike *SEP*, the *AP1*/*FUL* and *FLC* subfamilies have undergone severe rounds of structural divergence since the duplication of the ancestral gene. In addition to the insertion/deletion events that occurred in the ancestor of *AP1*/*FUL* and *FLC*, dramatic exon-intron structural changes, including exon loss, exonization, pseudoexonization, insertions, and deletions, have taken place in the respective ancestors of *FLC* and *AP1*/*FUL*. Divergence in gene structure of these two subfamilies resulted in shorter FLC-like proteins, but longer AP1/FUL-like proteins. Consistent with this, members of these two subfamilies tend to perform different functions in floral development. As has been reported, some *FLC* subfamily members act as floral repressors responsive to vernalization (Michaels and Amasino, 1999; Sheldon et al., 2006), while the *AP1*/*FUL*-like genes mainly function as positive regulators in determining the identities of inflorescences, floral meristems, and floral organs, and controlling the development of compound leaves and fruits (Irish and Sussex, 1990; Huijser et al., 1992; Gu et al., 1998; Pabón-Mora et al., 2012, 2013; Burko et al., 2013). Intriguingly, some *AP1*/*FUL* subfamily members are also involved in vernalization, such as *WAP1* in wheat (*Triticum aestivum*; Danyluk et al., 2003; Murai et al., 2003; Trevaskis et al., 2003; Yan et al., 2003; Kim et al., 2009). However, since members of other MADS-box gene subfamilies, such as *STMADS11*-like genes in grasses (Kane et al., 2005), are also identified as vernalization repressors, this type of function may have evolved multiple times independently. Frequent structural changes happened in the *AP1*/*FUL* subfamily may also be the cause of functional divergence between *AP1*/*FUL* and *SEP* subfamilies. We have recently revealed that the ancestral AP1/FUL protein lost the ability to interact with the AG and STK proteins in the MRCA of extant angiosperms (Li et al., 2015). This suggests that the two gene subfamilies have diverged at the early stage of angiosperm evolution, and that the functions of *AP1*/*FUL*-like genes further diversified during evolution due to the accumulation of more gene structural changes.

Different from the *SEP, AP1*/*FUL*, and *FLC* subfamilies, the *AGL6* subfamily originated before the diversification of extant seed plants, and experienced one round of gene duplication event in the MRCA of extant gymnosperms. In angiosperms, *AGL6*-like genes show various functions. For example, one of the *Arabidopsis AGL6*-like genes, *AGL6*, is responsible for the regulation of lateral organ development, flowering time, and circadian clock (Koo et al., 2010; Yoo et al., 2011; Huang et al., 2012, 2013), but the other one, *AGL13*, is involved in male and female gametophyte morphogenesis (Hsu et al., 2014). The *AGL6*-like gene in a basal eudicot species, *Nigella damascena*, acts as an A-function gene to determine the sepal and petal identities (Wang et al., 2015). In Zingiberales (monocot plants), the *AGL6*-like genes may regulate stamen morphology (Yockteng et al., 2013). Interestingly, in several angiosperm species, *AGL6*-like genes, such as *PhAGL6* of petunia (Rijpkema et al., 2009), *BEARDED-EAR* (*BDE*) of maize (Thompson et al., 2009), and *OsMADS6* of rice (Ohmori et al., 2009), function redundantly with *SEP*-like genes. In this article, we found that frequent structural change events have taken place during the evolution of angiosperm *AGL6*-like genes. Presumably, the unstable gene structures, plus regulatory divergence, have contributed to the functional diversification of angiosperm *AGL6*-like genes. Although some structural divergence events have also been revealed in the ancestor of angiosperm *AGL6*-like genes and the respective ancestors of gymnosperm Gg1 and Gg2 lineages, it seems that these ancestral proteins have similar interaction patterns. For instance, in gymnosperms, the AGL6-like proteins of *Gnetum gnemon*, GGM9 and GGM11, can interact with proteins of the *AP3*/*PI* and *AG*/*STK* subfamilies, and may have the ability to mediate multimeric protein complex formation (Wang et al., 2010). In the MRCA of extant angiosperms, AGL6 has relatively high possibility to interact with other floral proteins, similar to SEP (Li et al., 2015). Therefore, it is very likely that the quaternary complexes mediated by AGL6 have existed in the MRCA of extant seed plants (Wang et al., 2010). With the origin of SEP and the formation of obligate heterodimers between AP3 and PI in the MRCA of extant angiosperms (Melzer et al., 2014; Li et al., 2015), the multimerization of floral MADS-box proteins becomes equally dependent on SEP or AGL6. Afterwards, due to quick divergence of ancestral *SEP* and *AGL6* genes in exon-intron structure, together with point mutations and changes in expression regulation, the SEP-like proteins become major mediators of floral quartets in extant angiosperms. Overall, the evolution of the *SEP, AP1*/*FUL, FLC*, and *AGL6* subfamilies are complicated; their differences in exon-intron structures are only one aspect of their divergence. More studies are needed to clarify the functional diversification of these genes.

## Author contributions

XY, XD, RZ, XF, and LY analyzed data; XY, XD, RZ, GX, and HS wrote the paper; GX, HS, and HK designed the research.

### Conflict of interest statement

The authors declare that the research was conducted in the absence of any commercial or financial relationships that could be construed as a potential conflict of interest.

## Supplementary material

Table S1 **Genes used in this study**.

Dataset S1 **The phylogenetic tree used for gene classification**.

Dataset S2 **Matrices of the *AP1*/*FUL* subfamily. (A)** The matrix for phylogenetic analysis in consideration of exon-intron structural changes, in which only alignable sites are included. The resulting tree is shown in Figure S1. **(B)** The matrix based on an alignment generated by Probalign and the resulting tree. **(C)** The matrix for exon-intron structural change analysis.

Dataset S3 **Matrices of the *SEP1* subfamily. (A)** The matrix for phylogenetic analysis in consideration of exon-intron structural changes, in which only alignable sites are included. **(B)** The matrix based on an alignment generated by Probalign and the resulting tree. **(C)** The matrix for exon-intron structural change analysis.

Dataset S4 **Matrices of the *SEP3* subfamily. (A)** The matrix for phylogenetic analysis in consideration of exon-intron structural changes, in which only alignable sites are included. **(B)** The matrix based on an alignment generated by Probalign and the resulting tree. **(C)** The matrix for exon-intron structural change analysis.

Dataset S5 **Matrices of the *AGL6* subfamily. (A)** The matrix for phylogenetic analysis in consideration of exon-intron structural changes, in which only alignable sites are included. **(B)** The matrix based on an alignment generated by Probalign and the resulting tree. **(C)** The matrix for exon-intron structural change analysis.

Dataset S6 **Matrices of the *FLC* subfamily. (A)** The matrix for phylogenetic analysis in consideration of exon-intron structural changes, in which only alignable sites are included. **(B)** The matrix based on an alignment generated by Probalign and the resulting tree. **(C)** The matrix for exon-intron structural change analysis.

Dataset S7 **Matrices of the *SOC1* subfamily. (A)** The matrix for phylogenetic analysis in consideration of exon-intron structural changes, in which only alignable sites are included. **(B)** The matrix based on an alignment generated by Probalign and the resulting tree. **(C)** The matrix for exon-intron structural change analysis.

Dataset S8 **Matrices of the *AG/STK* subfamily. (A)** The matrix for phylogenetic analysis in consideration of exon-intron structural changes, in which only alignable sites are included. **(B)** The matrix based on an alignment generated by Probalign and the resulting tree. **(C)** The matrix for exon-intron structural change analysis.

Dataset S9 **Matrices for phylogenetic construction and exon-intron structural change inference among subfamilies. (A)** The matrix of alignment I and the resulting maximum likelihood tree. **(B)** The matrix of alignment II and the resulting maximum likelihood tree. The simplified trees are shown in Figure S8. **(C)** The matrix of alignment III. The resulting tree is shown in Figure 5. **(D)** The matrix for exon-intron structural change analysis among different subfamilies.

Figure S1 **Evolution of exon-intron structure in the *AP1/FUL* subfamily. (A)** A maximum-likelihood tree of the *AP1/FUL* subfamily, with higher-than-50% bootstrap values indicated for each node. Different mechanisms responsible for structural changes are marked on corresponding branches of the phylogenetic tree. Stars indicate structural changes involving non-triplet sequences. **(B)** Schematic representation of exon-intron structural changes. Exons and introns are represented by boxes and curved lines, respectively. Exon length is shown above the box, and intron length (if available) is indicated below the curved lines. Shared structural change events are linked by gray lines.

Figure S2 **Evolution of exon-intron structure in the *SEP1* subfamily. (A)** A maximum-likelihood tree of the *SEP1* subfamily. **(B)** Schematic representation of exon-intron structural changes. The symbols describing structural changes are the same as those in Figure S1.

Figure S3 **Evolution of exon-intron structure in the *SEP3* subfamily. (A)** A maximum-likelihood tree of the *SEP3* subfamily. **(B)** Schematic representation of exon-intron structural changes. The symbols describing structural changes are the same as those in Figure S1.

Figure S4 **Evolution of exon-intron structure in the *AGL6* subfamily. (A)** A maximum-likelihood tree of the *AGL6* subfamily. **(B)** Schematic representation of exon-intron structural changes. The symbols describing structural changes are the same as those in Figure S1.

Figure S5 **Evolution of exon-intron structure in the *FLC* subfamily. (A)** A maximum-likelihood tree of the *FLC* subfamily. **(B)** Schematic representation of exon-intron structural changes. Note that due to the dramatic sequence divergence of *OsMADS37*-like genes after gene duplication, the mechanisms underlying structural changes are difficult to determine. For these genes, only the exon-intron structures are shown. The symbols describing structural changes are the same as those in Figure S1.

Figure S6 **Evolution of exon-intron structure in the *SOC1* subfamily. (A)** A maximum-likelihood tree of the *SOC1* subfamily. **(B)** Schematic representation of exon-intron structural changes. The symbols describing structural changes are the same as those in Figure S1.

Figure S7 **Evolution of exon-intron structure in the *AG/STK* subfamily. (A)** A maximum-likelihood tree of the *AG/STK* subfamily. **(B)** Schematic representation of exon-intron structural changes. The symbols describing structural changes are the same as those in Figure S1.

Figure S8 **Simplified phylogenetic trees showing relationships of the *AP1*/*FUL, FLC, SEP*, and *AGL6* subfamilies, constructed based on alignments I (A) and II (B)**. The bootstrap values (>50%) obtained from maximum likelihood analysis and the posterior probabilities (>0.5) estimated by Bayesian inference are shown next to the nodes.

Figure S9 **Alignment of amino acids encoded by exon 7 of representatives of the *AP1/FUL, SEP, AGL6*, and *AG/STK* subfamilies**. Subfamily-specific motifs are highlighted by red boxes.

Figure S10 **Creation of the paleoAP1 motif**. Both nucleotide **(A)** and amino acid alignments **(B)** of the paleoAP1 motif in the sampled *AP1*/*FUL*-like genes and its corresponding regions in representatives of *SEP*-and *AGL6*-like genes are shown. On top of the alignments, an asterisk or a number indicates every ten nucleotides or amino acids. In **(A)**, coding sequences and 3′ untranslated regions are represented by uppercase and lowercase letters, respectively. In **(B)**, the paleoAP1 motif is boxed. Stars in the amino acid sequence correspond to stop codons.
