# Supplementary figures and images for: Prevalent Exon-Intron Structural Changes in the APETALA1/FRUITFULL, SEPALLATA, AGAMOUS-LIKE6, and FLOWERING LOCUS C MADS-Box Gene Subfamilies Provide New Insights into Their Evolution
Source: Front Plant Sci. 2016 May 2;7:598. doi: 10.3389/fpls.2016.00598 (PMC4852290; doi:10.3389/fpls.2016.00598)

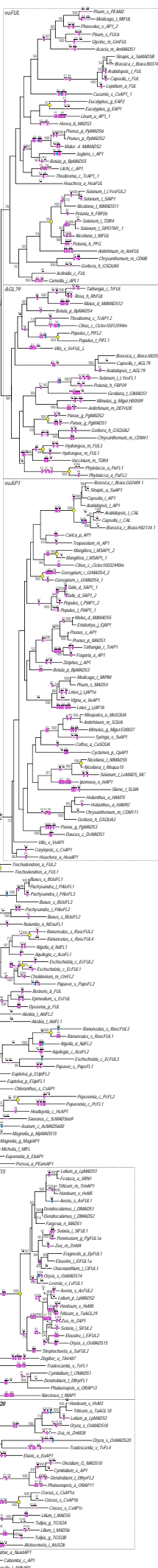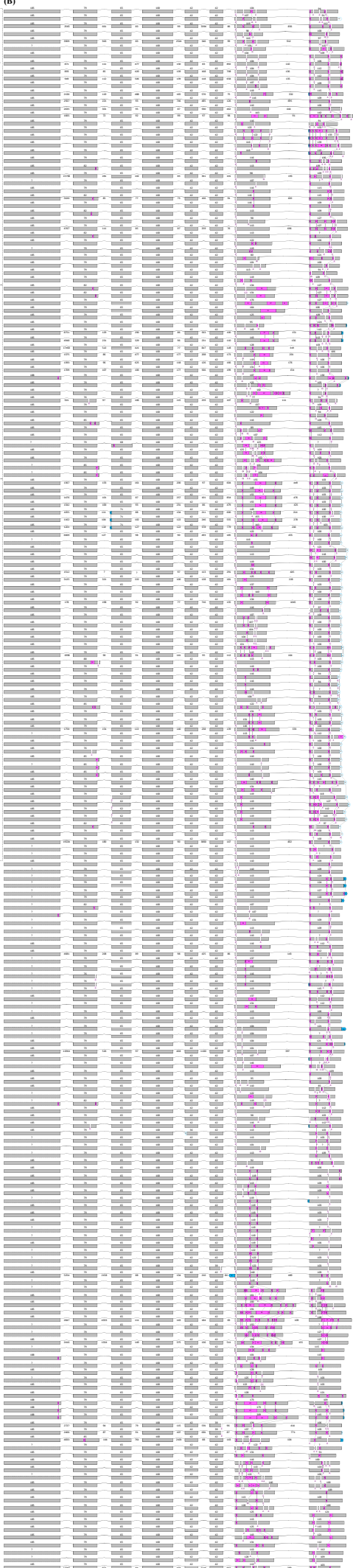

Outgroups

Legend: Green, Blue, Red, Purple, Orange, Yellow

Supplement: Figure S1 — Evolution of exon-intron structure in the AP1/FUL subfamily. (A) A maximum-likelihood tree of the AP1/FUL subfamily, with higher-than-50% bootstrap values indicated for each node. Different mechanisms responsible for structural changes are marked on corresponding branches of the phylogenetic tree. Stars indicate structural changes involving non-triplet sequences. (B) Schematic representation of exon-intron structural changes. Exons and introns are represented by boxes and curved lines, respectively. Exon length is shown above the box, and intron length (if available) is indicated below the curved lines. Shared structural change events are linked by gray lines. [file Image1.PDF]

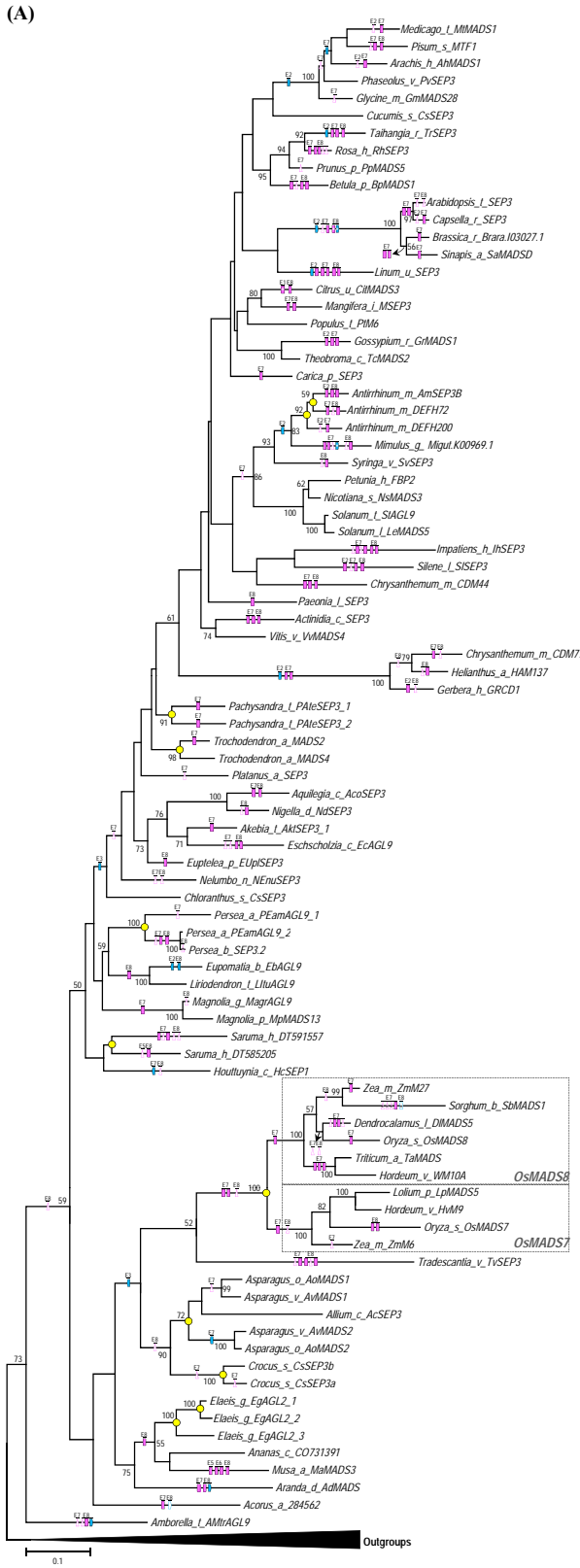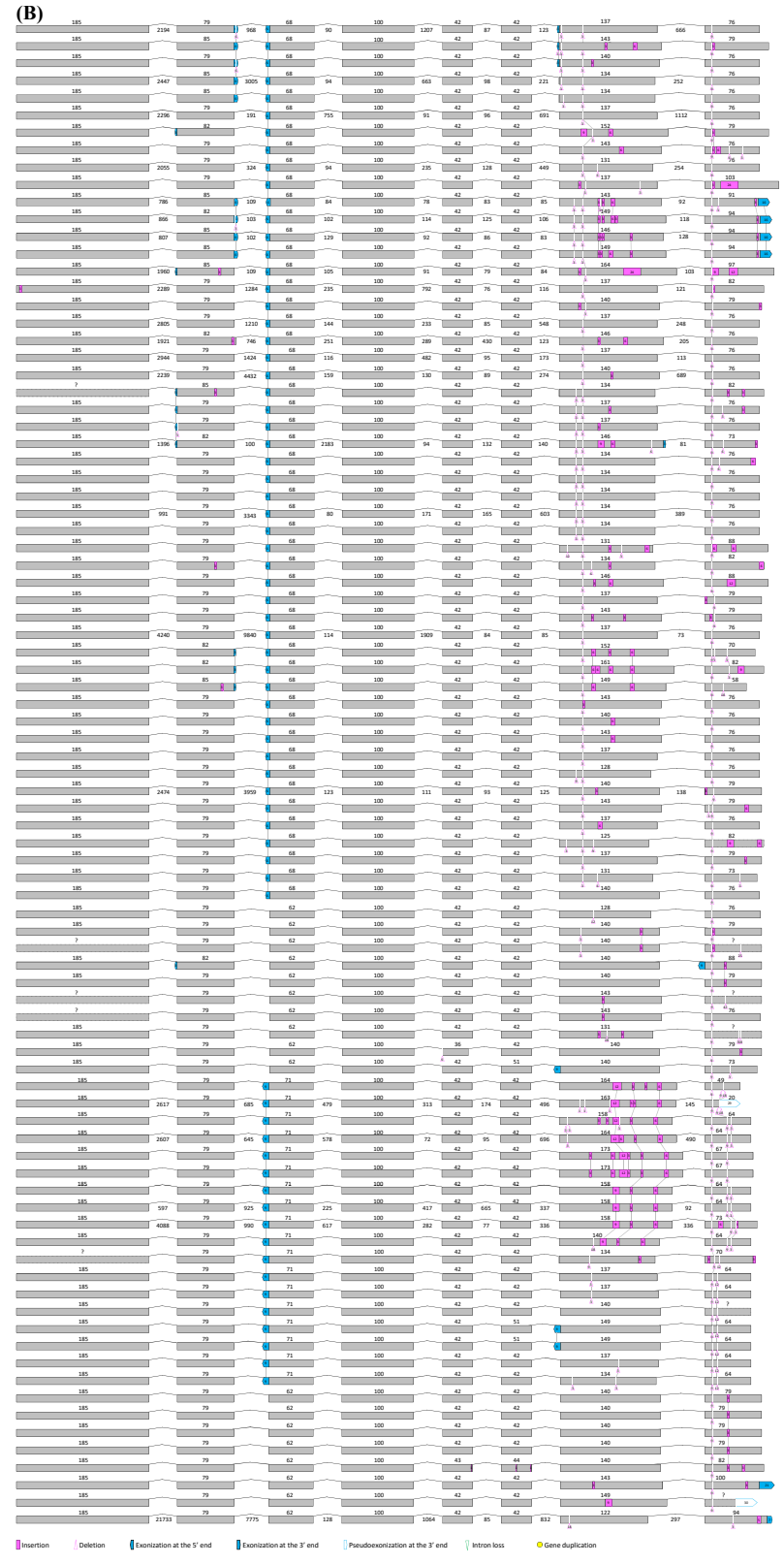

Supplement: Figure S3 — Evolution of exon-intron structure in the SEP3 subfamily. (A) A maximum-likelihood tree of the SEP3 subfamily. (B) Schematic representation of exon-intron structural changes. The symbols describing structural changes are the same as those in Figure S1. [file Image3.PDF]

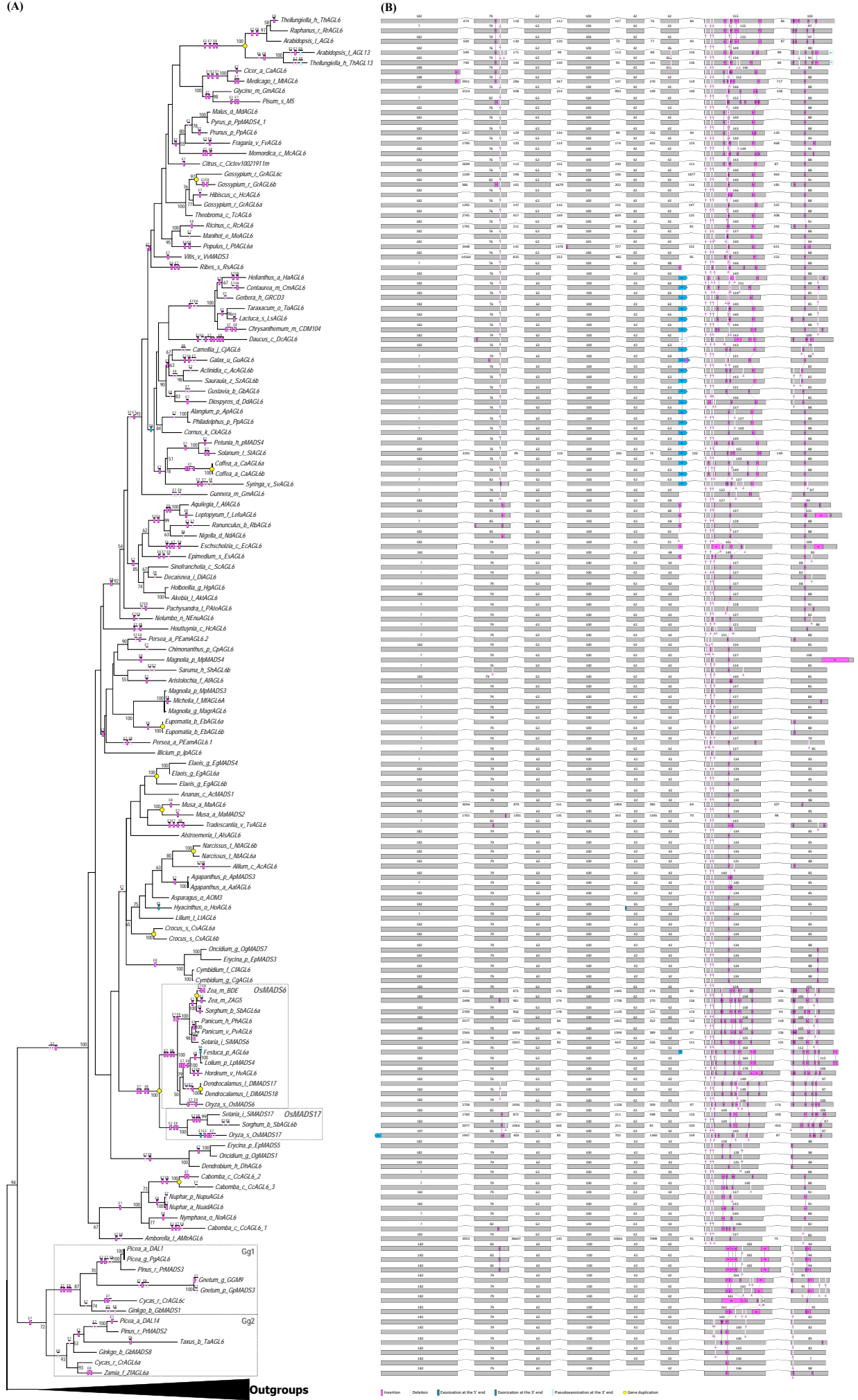

Supplement: Figure S4 — Evolution of exon-intron structure in the AGL6 subfamily. (A) A maximum-likelihood tree of the AGL6 subfamily. (B) Schematic representation of exon-intron structural changes. The symbols describing structural changes are the same as those in Figure S1. [file Image4.PDF]

(A)

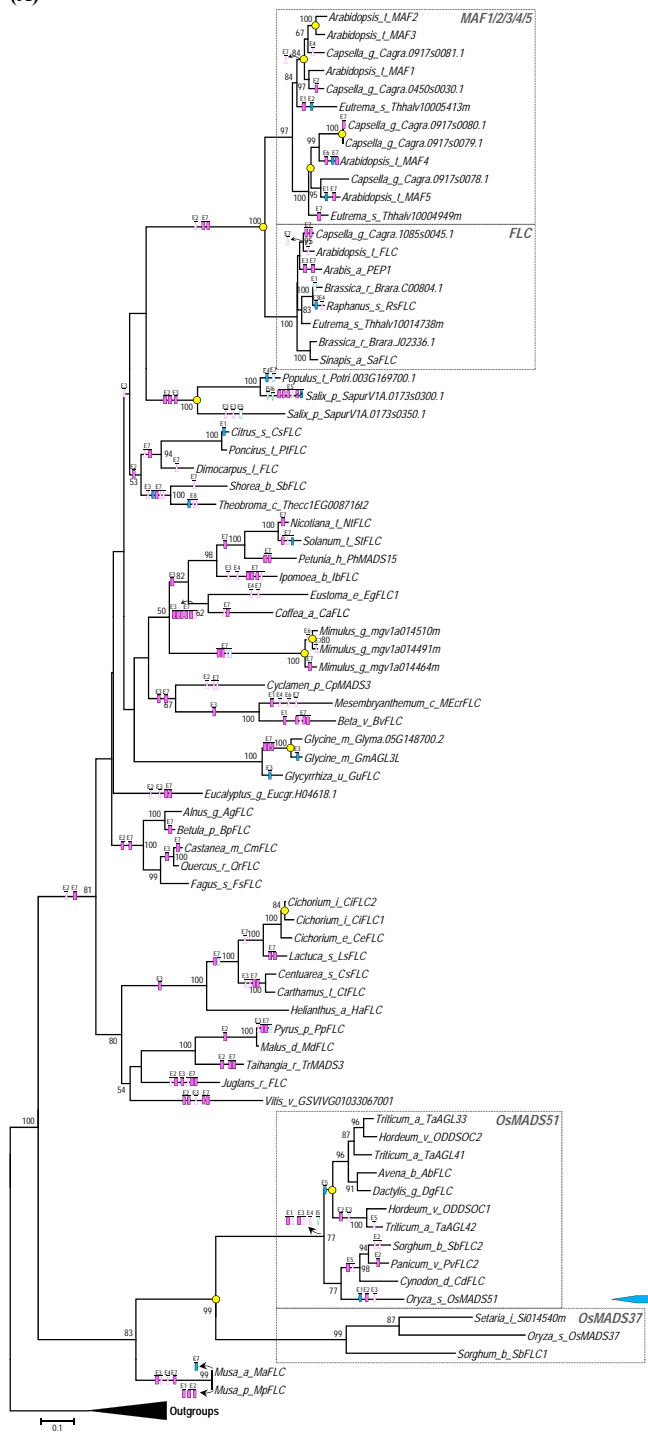

(B)

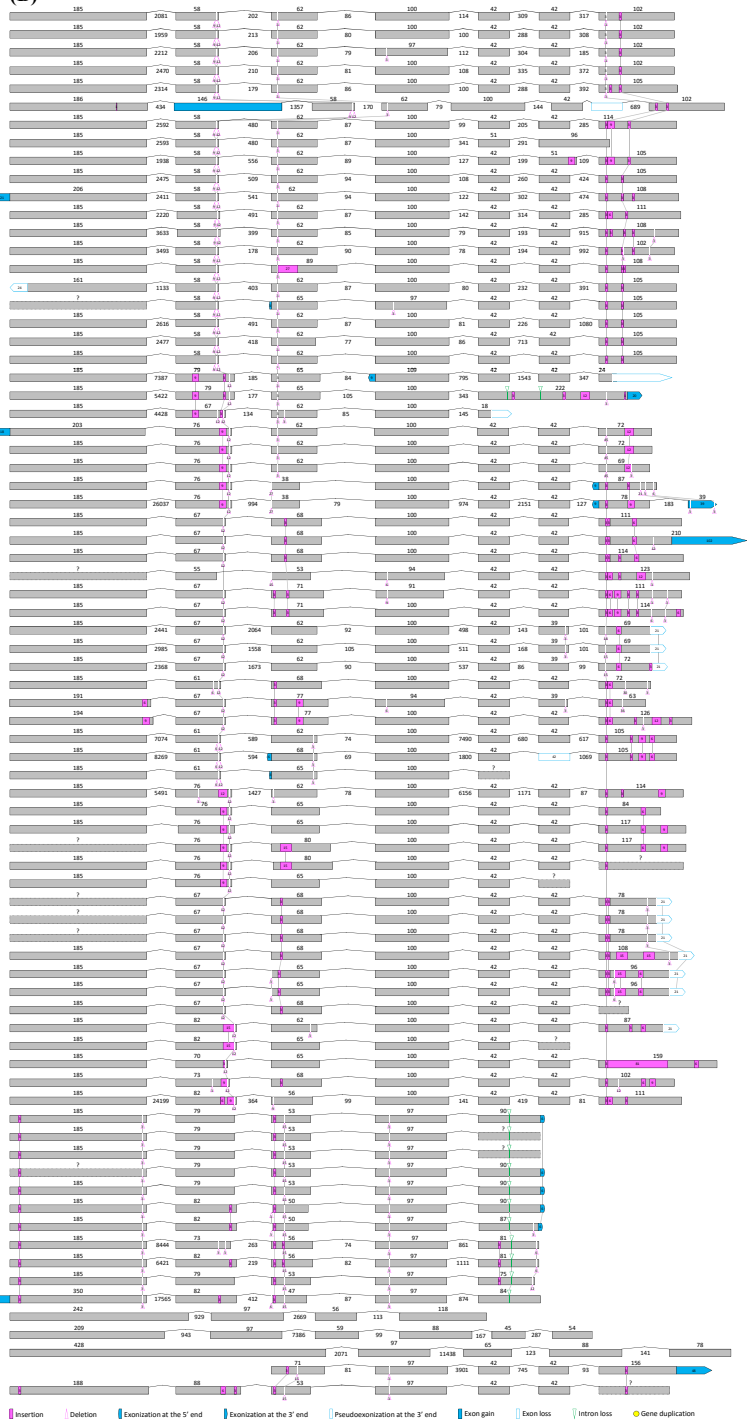

Supplement: Figure S5 — Evolution of exon-intron structure in the FLC subfamily. (A) A maximum-likelihood tree of the FLC subfamily. (B) Schematic representation of exon-intron structural changes. Note that due to the dramatic sequence divergence of OsMADS37-like genes after gene duplication, the mechanisms underlying structural changes are difficult to determine. For these genes, only the exon-intron structures are shown. The symbols describing structural changes are the same as those in Figure S1. [file Image5.PDF]

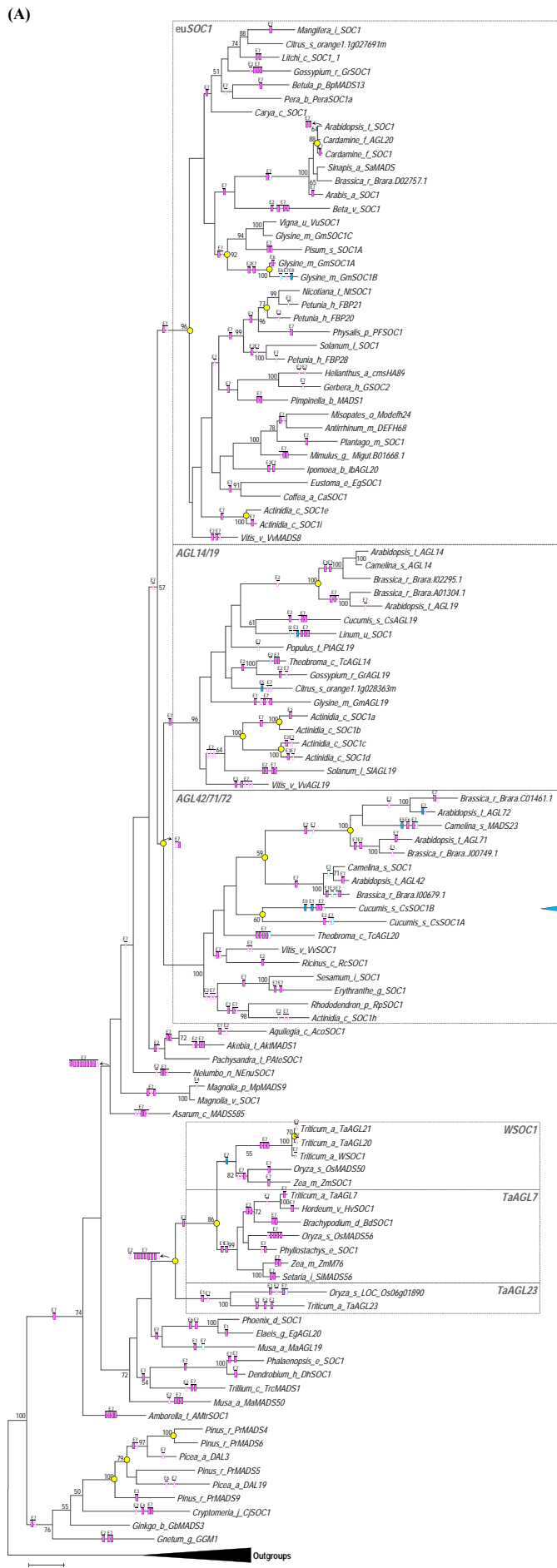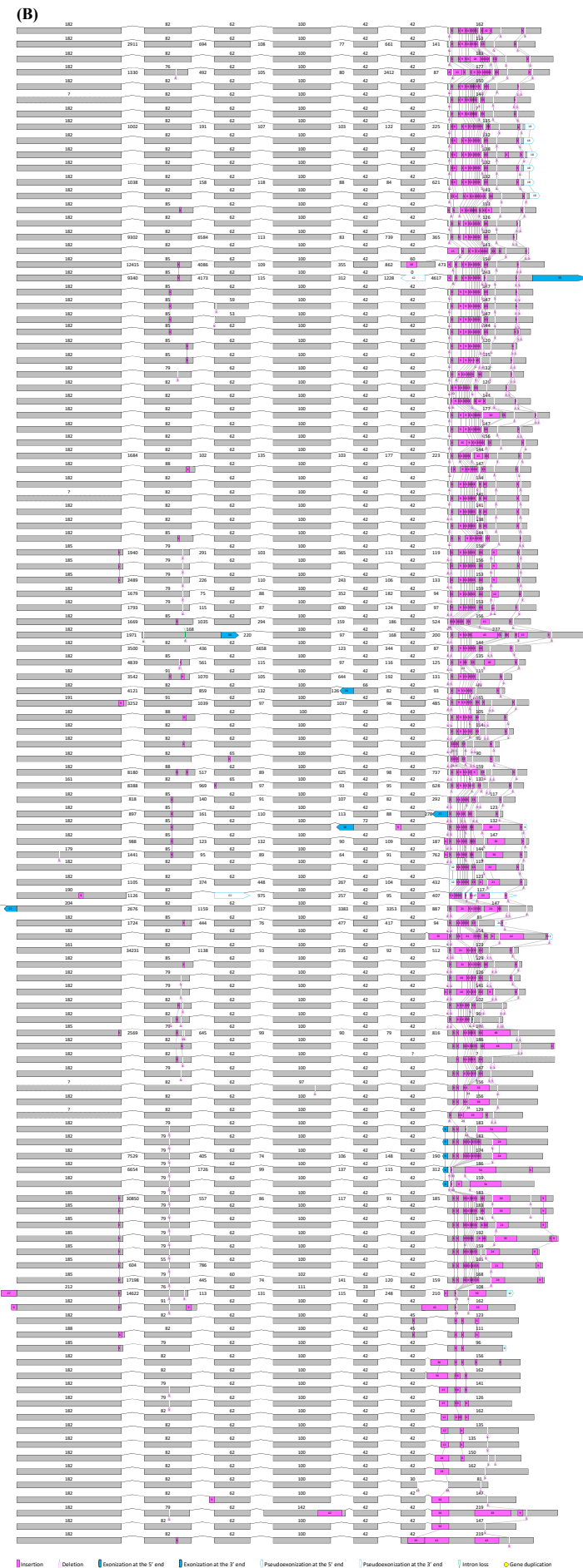

Supplement: Figure S6 — Evolution of exon-intron structure in the SOC1 subfamily. (A) A maximum-likelihood tree of the SOC1 subfamily. (B) Schematic representation of exon-intron structural changes. The symbols describing structural changes are the same as those in Figure S1. [file Image6.PDF]

(A)

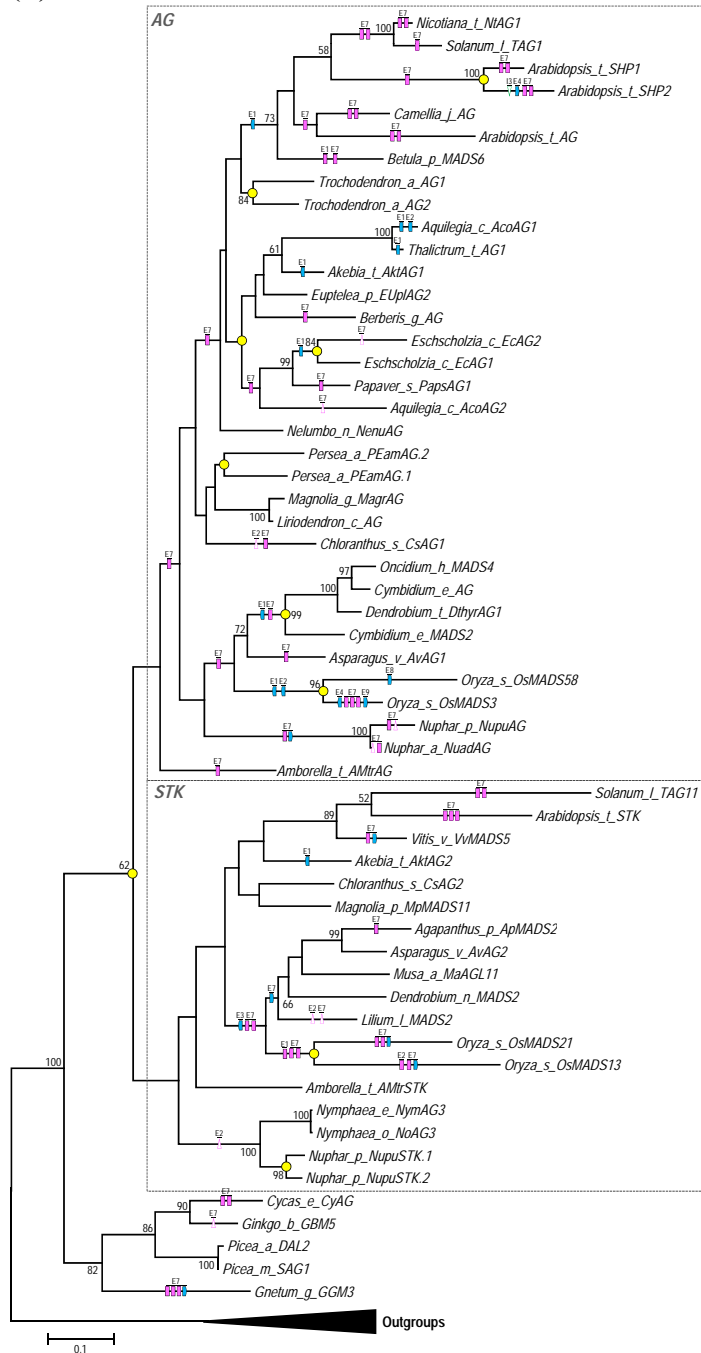

(B)

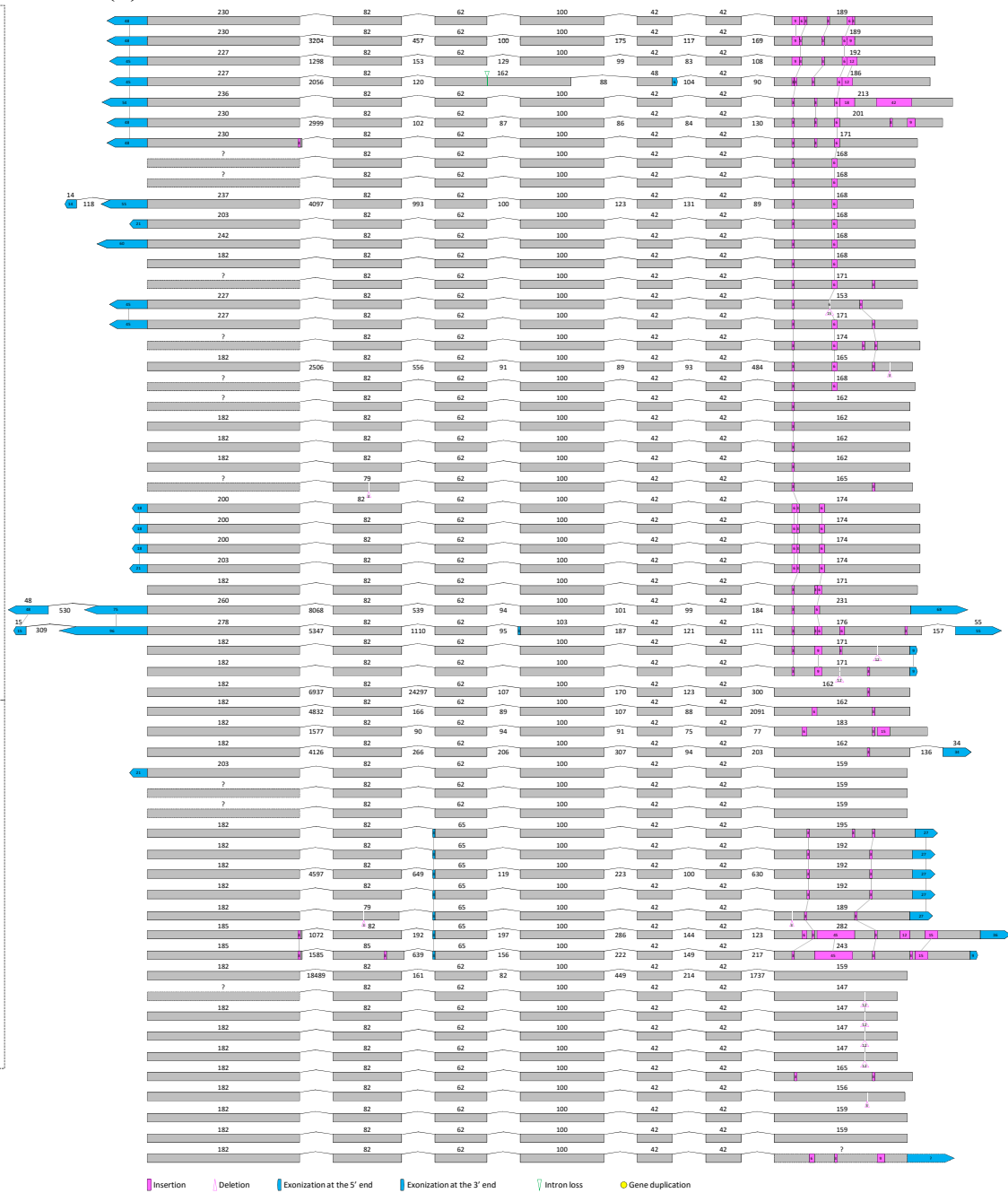

Supplement: Figure S7 — Evolution of exon-intron structure in the AG/STK subfamily. (A) A maximum-likelihood tree of the AG/STK subfamily. (B) Schematic representation of exon-intron structural changes. The symbols describing structural changes are the same as those in Figure S1. [file Image7.PDF]

**(A)**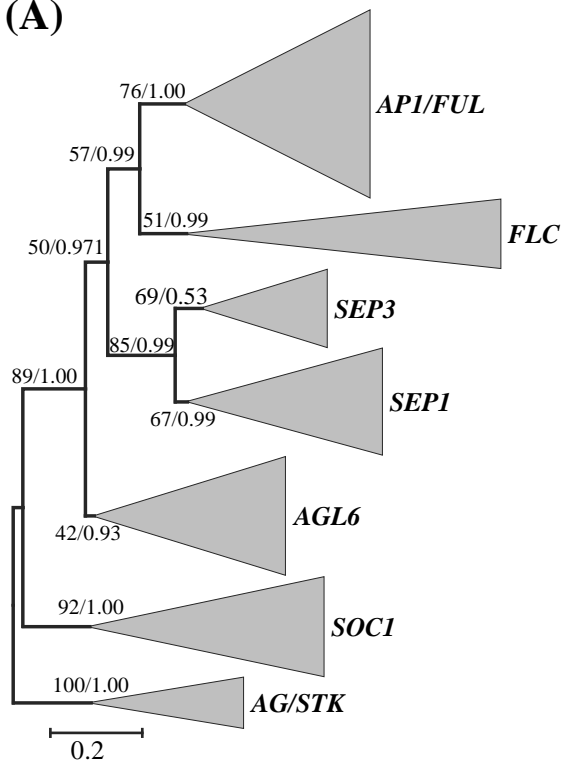**(B)**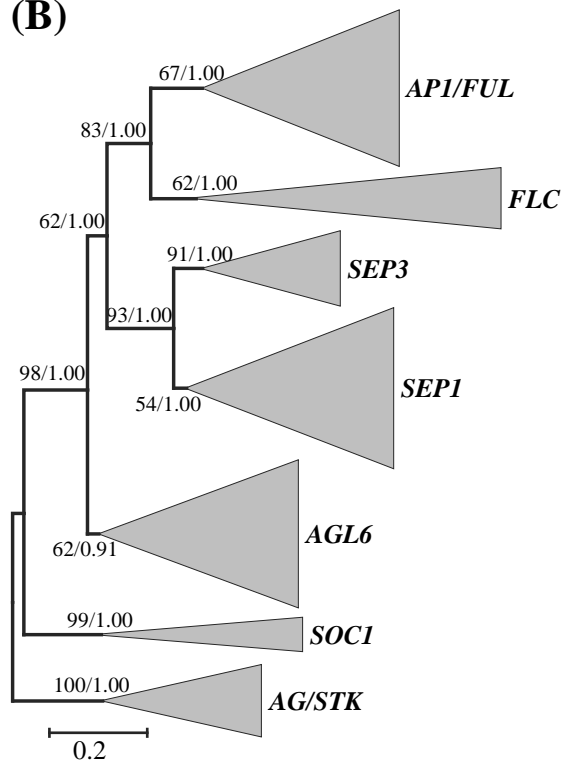

Supplement: Figure S8 — Simplified phylogenetic trees showing relationships of the AP1/FUL, FLC, SEP, and AGL6 subfamilies, constructed based on alignments I (A) and II (B). The bootstrap values (>50%) obtained from maximum likelihood analysis and the posterior probabilities (>0.5) estimated by Bayesian inference are shown next to the nodes. [file Image8.PDF]
