# Supplementary material for: Prevalent Exon-Intron Structural Changes in the APETALA1/FRUITFULL, SEPALLATA, AGAMOUS-LIKE6, and FLOWERING LOCUS C MADS-Box Gene Subfamilies Provide New Insights into Their Evolution
Source: Front Plant Sci. 2016 May 2;7:598. doi: 10.3389/fpls.2016.00598 (PMC4852290; doi:10.3389/fpls.2016.00598)
Supplement: Figure S9 — Alignment of amino acids encoded by exon 7 of representatives of the AP1/FUL, SEP, AGL6, and AG/STK subfamilies. Subfamily-specific motifs are highlighted by red boxes. [file Image9.PDF]

```
Aquilegia_c_AcoFL1      --L--KEK--EK--EL--AQQ-SP------------------------WEQPN--R-----------------GQNSPS--MLIS-QTLPSLNIG
Aquilegia_c_AcoFL2      --I--KEK--EL--EL------SL------------------------HEHID--D-----------------VETSPS--L-PS-ERLPVLNIS
Magnolia_g_MagrAP1      --I--QEK--EK--AM--AQQ-AQ------------------------WEQQN--Q-----------------SQSSPS--FLLA-SPLPTLNIG
Nuphar_a_NuadAP1        --L--REQEAAL--NC--LQS-AR------------------------SE-----Q-----------------PQSRTVG-FLFN-SHSPTPDSG
Amborella_t_AMtrAP1     --IVAKEK--AL-AQA--QAT-TH------------------------WE-----Q-----------------EETAH---FLSTSHPHPALNVR
                                                                                                   FUL motif

Aquilegia_c_AcoSEP3     --L--EEG--TQ--PN--HHH---------------------------WDPNM--HNGVT-FAR-Q----Q-AQAQGEG--FFHPLECEPTLQIG
Chloranthus_s_CsSEP3    --L--DEV--TP--AN--PHQ-G-------------------------WDPNP--H-GVS-YGR-Q----A-AQQQGDG--FFHPLECEPTLQIG
Magnolia_g_MagrAGL9     --L--EEG--AQ--AN--HNQ-V-------------------------WEPNA--H-AVDSYNR-Q----Q-PQQQGDG--FFHPLECEPTLHIG
Amborella_t_AMtrAGL9    --L--EAA------------G-G-------------------------WDSTG--H-QME-YNR-Q----P-AQAQADN--FFHPLECDPTLQIG
                                                                                                   SEP I motif

Aquilegia_c_AcoSEP1     --L--DEK--AAD-SHDPLQL-L-------------------------WEAGH--K-HTP-FNH-QTQ--T-LQ--PER--FFQPLECNSTMQIR
Aquilegia_c_AcoSEP2A    --L--DER--IAE-NA--LRL-P-------------------------WASGE--Q-NIP-YCR-Q----P-AQ--SEE--FFQPLGCNSTLHVG
Magnolia_g_MagrAGL2     --L--EES--GRE-NL--LQL-S-------------------------WDTGA--Q-NMSSYNR-Q----P-SN--YEG--FFQPLDCQPTLQMG
Nuphar_a_NuadAGL2       --L--EGAA-GSA-NH--QQL-S-------------------------WEN------GGQ-HLQ-YGRHSG-PQ--KDG--FYHPLECDSTLQIG
Amborella_t_AMtrAGL2    --L--EGA--SAS-NP--PQL-A-------------------------WENNG--Q-NIH-YNR-Q----P-AH--TEG--FFHPLECDSTLQIG
                                                                                                   SEP I motif

Aquilegia_f_AfAGL6      YQL---DA--EGQ-AP--YRA-LQ---GS-------------------WESNALVA-SNN-FSM-H----A-SQ--SSS------MDCEPTLQIG
Magnolia_p_MpMADS3      --L---EA--KGQ-GA--FRA-MQ---AS-------------------WESGPLVG-NNG-FPM-H----P-SQ--SAA------IECEPTLQIG
Magnolia_p_MpMADS4      --L---EA--EGQ-GP--FRC-IQ---GS-------------------WESGAMVG-NNN-FSM-N----A-PQ--AAP------MECEPTLQIG
Nuphar_p_NupuAGL6       --L---ES--QG--HV--FRS-MP---GSSS-----------------WESG-VVG-NNS-LNM-N----A-AQ--ANH------IDCQPTLQIG
Amborella_t_AMtrAGL6    --L---ES--DGQ-GS--FRG-IQ---GT-------------------WESGTVVG-NNA-FAV-N----P-SH--ANP------IDCEPTLQIG
Picea_a_DAL1            --L--SET--EGR-DVI-TGI-EQ---TSNTNTGTNGP----------WDS-S-IT-NTA-YAL-S----H-PQ--QNSNASLHHVDCEPTLQIG
Ginkgo_b_GbMADS1        --L--SES--EGR-NAT-HDM-RH---PTDDN--------------GPWNP-S-VN-GG--YAL--------PS--TQQNTNLHPVDCEPTLQIG
Cycas_r_CrAGL6a         --L--SEA--EGQ-NAA-FNA-MQ---AT-----------------GSWDSNA-VA-NNA-YAL-Q----P-NQ--ANA------VDCEPTLQIG
Picea_a_DAL14           --L--QEA--EGQ--A--FNS-MQ---APPP---------------HAWDSHA-VA-NNA-YAM-Q------HQ--SNA------VDCEPTLQIG
Ginkgo_b_GbMADS8        --L--SEA--EEQ-RA--FSA-MQ---DP-----------------GSWDSNA-VA-NNA-YAM-P----P-NQ--SNA------VDCEPTLQIG
Cycas_r_CrAGL6c         --L--SES--EGR-NA--VNA-MHN--TTIATIDNNNSNNDNNN--VPWDSSA-VS-SGA-YGL------------------------QPTLQIG
                                                                                                      AGL6 I motif

Aquilegia_c_AcoAG1      --I--AAN--ERAPEH--MNL-MP---AN--------EYHALSSAP--FDSRN-FM-PAN-LLD-H----N-NN--YSR------SDQ-TTLQLG
Akebia_t_AktAG1         --I--AEN--ERAGQH--MNL-MP---GN--------EYEVMSSAP--FDSRN-FL-QVN-LLE-P----N-NH--YSH------TDQ-TALQLG
Aquilegia_c_AcoAG2      --I--TAN--ERAQQH--MNS-LP---GN--------VYEAITSAP--YNSRD-FL-QVN-LRESK----P-NQ--YC--------DS-TALQLC
Nelumbo_n_NenuAG        --I--AEN--ERAQQQ--MSM-IP---AS--------EYEVMPPQS--FDSRN-FL-QVN-LLE-P----N-HH--YSR------QEQ-TALQLG
Magnolia_g_MagrAG       --I--TEN--ERAQQQ--MGM-LP---PP--------EYDVMP--G--FDSRN-FL-QVN-LMD-S----S-HH--YSH------QEQ-TALQLG
Chloranthus_s_CsAG1     --I--AEN--ERAQQH--MNV-LP---GP--------EYDVMP--A--FDGRN-FL-PVN-LLG-S----NHHQ--FSH------QDQ-TALQLG
Nuphar_p_NupuAG         --I--SEN--ERA-HQ--QQH-ISMMAGPS-------EYELLPT-T--FQHVN-LL-E--------P----SHHH--YSH------QER-TALQLG
Amborella_t_AMtrAG      --I--AEN--ERA-QH--MNM-LP---GP--------EYDVLP--P--FDSRN-YL-QVN-LLE-P----NHHN--YSH------QEQ-TALQLG
Akebia_t_AktAG2         --I--AEN--ENA-QQ--TSM-VP---AQ--------EFDAIQ--T--FDSRN-YF-QMN-MLE-GG---A--A--YSH------ADQ-TALHLG
Chloranthus_s_CsAG2     --I--AEN--ENA-QQ--ANM-LP---GP--------EFDTLP--T--FDSRN-YF-QAN-ILE-AA---P--Q--YSH------QDQ-TALHLG
Magnolia_p_MpMADS11     --I--AEN--ERA-QQ--ANV-LP---AP--------EFDTLP--S--FDSRN-YF-EAN-MLE-AA---S--H--YSH------QDQ-TALHLG
Nuphar_p_NupuSTK.1      --V--AES--ERA-QQ--SNM-LP---GS--------DYEAMN--T--FDSRN-FF-PVN-MIH------------YSN------QDQ-TALHLG
Amborella_t_AMtrSTK     --I--AEQ--DRT-QQ--SNM-LP---AP--------EFDAIP--T--FDSQN-YY-QVN-LME-G----A-HH--YRN------QDQ-TALHLG
Nuphar_p_NupuSTK.2      --V--AES--ERA-EQ--SNM-LP---GS--------DYEAMQ--T--FDSRN-FF-PVN-MLH------------YSN------QDQ-TALHLG
Picea_a_DAL2            --I--AEC--QNS-HN--TNM-LS---AP--------EYDALP--A--FDSRN-FL-HAN-LID-A----A-HH--YAH------QEQ-TTLQLG
Ginkgo_b_GBM5           --I--AEC--ESS-QN--ANM-LP---GP--------EFDALP--G--FDSRH-FL-HAS-IMD-A------HH--YAQ------QDQ-TALQLG
Cycas_e_CyAG            --I--AEY--ESN-QN--TNVLIP---GP--------EFDALP--A--FDSRN-FL-HAN-LIE-A----AAHH--YTQ------QDQ-AALQLG
                                                                   AG I motif                                AG II motif
```
