# Supplementary material for: Prevalent Exon-Intron Structural Changes in the APETALA1/FRUITFULL, SEPALLATA, AGAMOUS-LIKE6, and FLOWERING LOCUS C MADS-Box Gene Subfamilies Provide New Insights into Their Evolution
Source: Front Plant Sci. 2016 May 2;7:598. doi: 10.3389/fpls.2016.00598 (PMC4852290; doi:10.3389/fpls.2016.00598)
Supplement: Figure S10 — Creation of the paleoAP1 motif. Both nucleotide (A) and amino acid alignments (B) of the paleoAP1 motif in the sampled AP1/FUL-like genes and its corresponding regions in representatives of SEP-and AGL6-like genes are shown. On top of the alignments, an asterisk or a number indicates every ten nucleotides or amino acids. In (A), coding sequences and 3′ untranslated regions are represented by uppercase and lowercase letters, respectively. In (B), the paleoAP1 motif is boxed. Stars in the amino acid sequence correspond to stop codons. [file Image10.PDF]

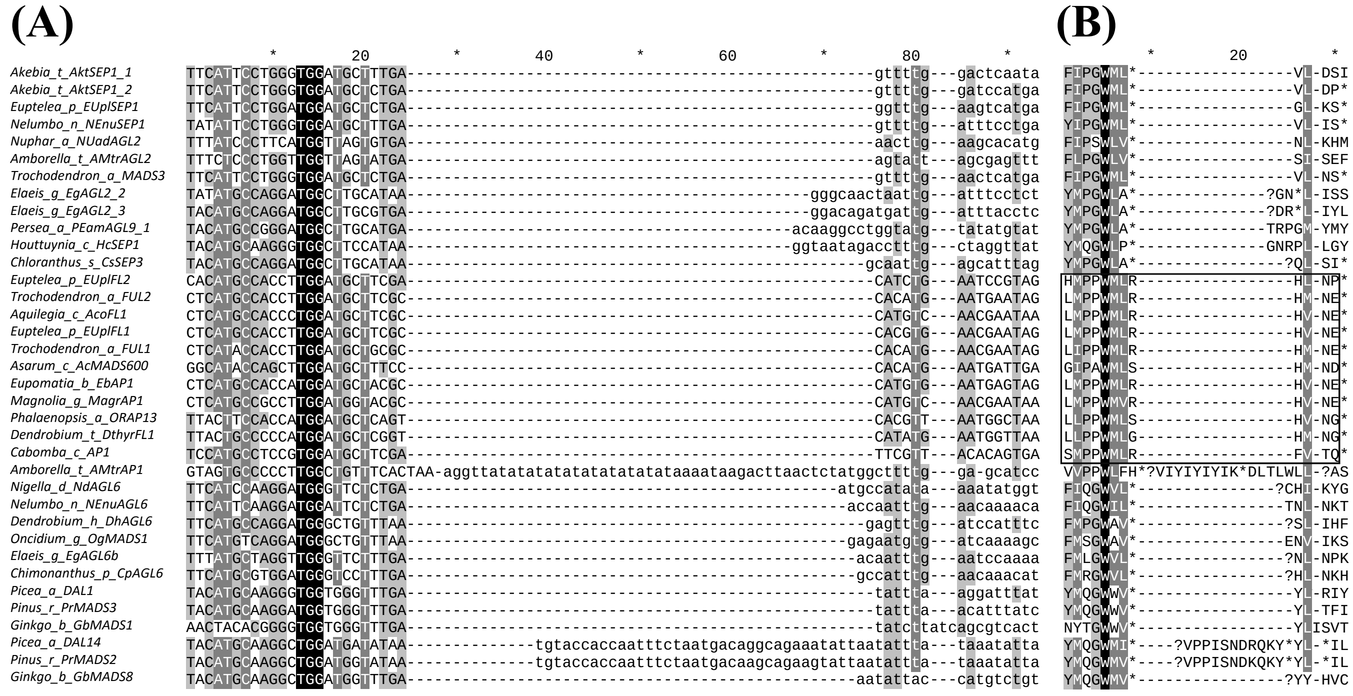

(A)
* 20 * 40 * 60 * 80 *
Akebia_t_AktSEP1_1 TTCATTCCTGGGTGGATGCTTTGA--gtttg---gactcaata
Akebia_t_AktSEP1_2 TTCATTCCTGGGTGGATGCTCTGA--gtttg---gatccatga
Euptelea_p_EUplSEP1 TTCATTCCTGGATGGATGCTCTGA---ggtttg---aagtcatga
Nelumbo_n_NEnuSEP1 TATATTCCTGGGTGGATGCTTTGA--gtttg---atttcctga
Nuphar_a_NUadAGL2 TTTATCCCTTCATGGTTAGTGTGA---aacttg---aagcacatg
Amborella_t_AMtrAGL2 TTTCTCCCTGGTTGGTTAGTATGA---agtatt---agcgagttt
Trochodendron_a_MADS3 TTCATTCCTGGGTGGATGCTCTGA--gtttg---aactcatga
Elaeis_g_EgAGL2_2 TATATGCCAGGATGGCTTGCATAA--gggcaactaattg---atttcctct
Elaeis_g_EgAGL2_3 TACATGCCAGGATGGCTTGCGTAA--ggacagatgattg---atttacctc
Persea_a_PEamAGL9_1 TACATGCCGGGATGGCTTGCATGA--acaaggcctggtatg---tatatgtat
Houttuynia_c_HcSEP1 TACATGCAAGGGTGGCTTCCATAA---ggtaatagaccttg---ctaggttat
Chloranthus_s_CsSEP3 TACATGCCAGGATGGCTTGCATAA--gcaattg---agcatttag
Euptelea_p_EUplFL2 CACATGCCACCTTGGATGCTTCGA---CATCTG---AATCCGTAG
Trochodendron_a_FUL2 CTCATGCCACCTTGGATGCTTCGC---CACATG---AATGAATAG
Aquilegia_c_AcoFL1 CTCATGCCACCCTGGATGCTTCGC---CATGTC---AACGAATAA
Euptelea_p_EUplFL1 CTCATGCCACCTTGGATGCTTCGC---CACGTG---AACGAATAG
Trochodendron_a_FUL1 CTCATACCACCTTGGATGCTGCGC---CACATG---AACGAATAG
Asarum_c_AcMADS600 GGCATACCAGCTTGGATGCTTTCC---CACATG---AATGATTGA
Eupomatia_b_EbAP1 CTCATGCCACCATGGATGCTACGC---CATGTG---AATGAGTAG
Magnolia_g_MagrAP1 CTCATGCCGCCTTGGATGGTACGC---CATGTC---AACGAATAA
Phalaenopsis_a_ORAP13 TTACTTCCACCATGGATGCTCAGT---CACGTT---AATGGCTAA
Dendrobium_t_DthyrFL1 TTACTGCCCCCATGGATGCTCGGT---CATATG---AATGGTTAA
Cabomba_c_AP1 TCCATGCCTCCGTGGATGCTTCGA---TTCGTT---ACACAGTGA
Amborella_t_AMtrAP1 GTAGTGCCCCCTTGGCTGTTCACTAA-aggttatatatatatatatatatataaaataagacttaactctatggcttttg---ga-gcatcc
Nigella_d_NdAGL6 TTCATCCAAGGATGGGTTCTCTGA---atgccatata---aaatatggt
Nelumbo_n_NEnuAGL6 TTCATTCAAGGATGGATTCTCTGA--accaatttg---aacaaaaca
Dendrobium_h_DhAGL6 TTCATGCCAGGATGGGCTGTTTAA--gagtttg---atccatttc
Oncidium_g_OgMADS1 TTCATGTCAGGATGGGCTGTTTAA--gagaatgtg---atcaaaagc
Elaeis_g_EgAGL6b TTTATGCTAGGTTGGGTTCTTTGA---acaatttg---aatccaaaa
Chimonanthus_p_CpAGL6 TTCATGCGTGGATGGGTCCTTTGA---gccatttg---aacaaacat
Picea_a_DAL1 TACATGCAAGGGTGGTGGGTTTGA---tattta---aggatttat
Pinus_r_PrMADS3 TACATGCAAGGATGGTGGGTTTGA---tattta---acatttatc
Ginkgo_b_GbMADS1 AACTACACGGGGTGGTGGGTTTGA---tatcttatcagcgtcact
Picea_a_DAL14 TACATGCAAGGCTGGATGATATAA--------------tgtaccaccaatttctaatgacaggcagaaatattaatattta---taaatatta
Pinus_r_PrMADS2 TACATGCAAGGCTGGATGGTATAA--------------tgtaccaccaatttctaatgacaagcagaagtattaatattta---taaatatta
Ginkgo_b_GbMADS8 TACATGCAAGGCTGGATGGTTTGA---aatattac---catgtctgt
(B)
* 20 *
FIPGWML*-----------------VL-DSI
FIPGWML*-----------------VL-DP*
FIPGWML*-----------------GL-KS*
YIPGWML*-----------------VL-IS*
FIPSWLV*-----------------NL-KHM
FLPGWLV*-----------------SI-SEF
FIPGWML*-----------------VL-NS*
YMPGWLA*--------------?GN*L-ISS
YMPGWLA*--------------?DR*L-IYL
YMPGWLA*--------------TRPGM-YMY
YMQGWLP*--------------GNRPL-LGY
YMPGWLA*----------------?QL-SI*
HMPPWMLR-----------------HL-NP*
LMPPWMLR-----------------HM-NE*
LMPPWMLR-----------------HV-NE*
LMPPWMLR-----------------HV-NE*
LIPPWMLR-----------------HM-NE*
GIPAWMLS-----------------HM-ND*
LMPPWMLR-----------------HV-NE*
LMPPWMVR-----------------HV-NE*
LLPPWMLS-----------------HV-NG*
LLPPWMLG-----------------HM-NG*
SMPPWMLR-----------------FV-TQ*
VVPPWLFH*?VIYIYIYIK*DLTLWLL-?AS
FIQGWVL*---------------?CHI-KYG
FIQGWIL*----------------TNL-NKT
FMPGWAV*----------------?SL-IHF
FMSGWAV*----------------ENV-IKS
FMLGWVL*----------------?NL-NPK
FMRGWVL*----------------?HL-NKH
YMQGWWV*-----------------YL-RIY
YMQGWWV*-----------------YL-TFI
NYTGWWV*-----------------YLISVT
YMQGWMI*----?VPPISNDRQKY*YL-*IL
YMQGWMV*----?VPPISNDKQKY*YL-*IL
YMQGWMV*----------------?YY-HVC
