# Supplementary material for: Prevalent Exon-Intron Structural Changes in the APETALA1/FRUITFULL, SEPALLATA, AGAMOUS-LIKE6, and FLOWERING LOCUS C MADS-Box Gene Subfamilies Provide New Insights into Their Evolution
Source: Front Plant Sci. 2016 May 2;7:598. doi: 10.3389/fpls.2016.00598 (PMC4852290; doi:10.3389/fpls.2016.00598)
Supplement: Figure S2 — Evolution of exon-intron structure in the SEP1 subfamily. (A) A maximum-likelihood tree of the SEP1 subfamily. (B) Schematic representation of exon-intron structural changes. The symbols describing structural changes are the same as those in Figure S1. [file Image2.PDF]

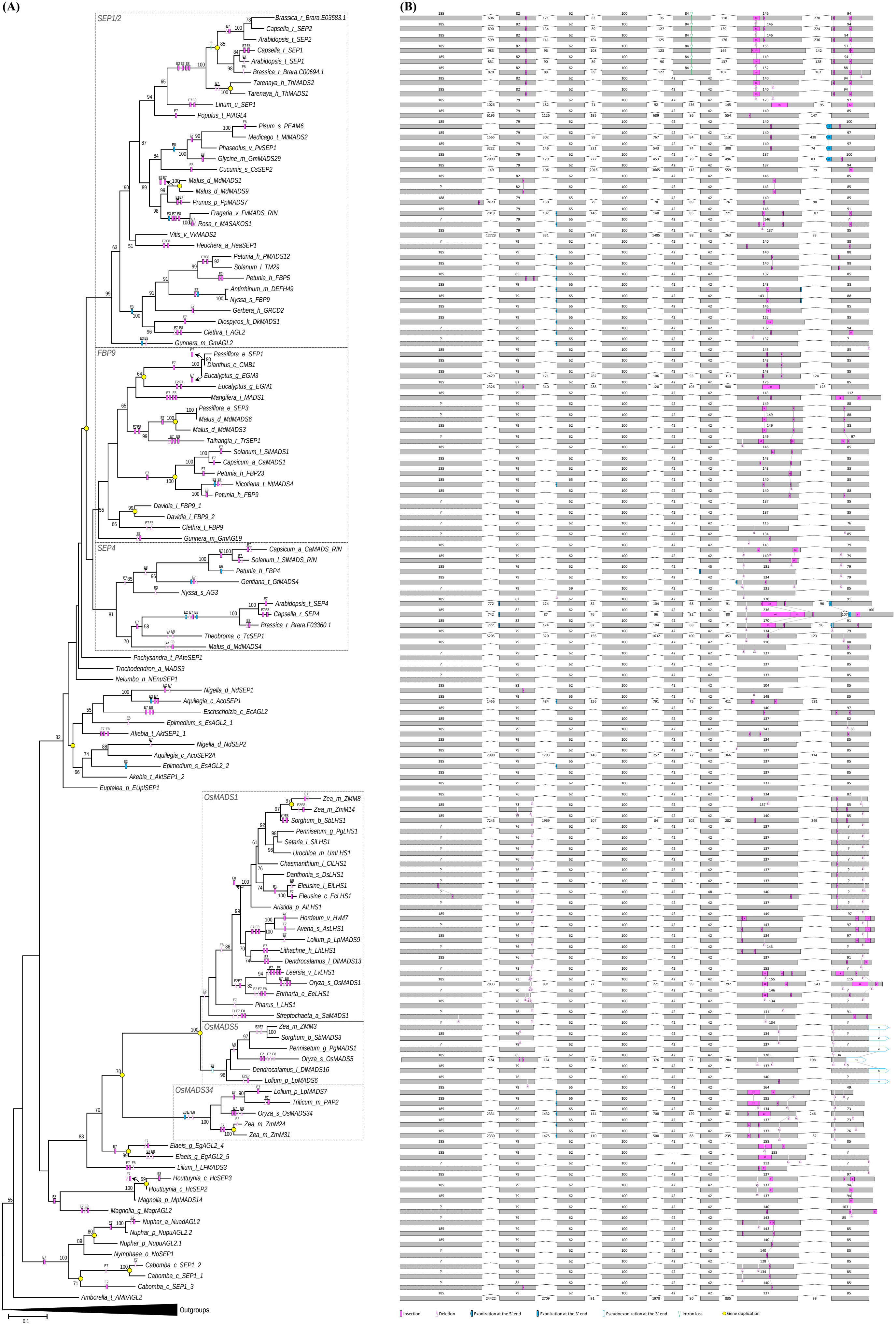

(A)
(B)
SEP1/2
Brassica_r_Brara.E03583.1
Capsella_r_SEP2
Arabidopsis_t_SEP2
Capsella_r_SEP1
Arabidopsis_t_SEP1
Brassica_r_Brara.C00694.1
Tarenaya_h_ThMADS2
Tarenaya_h_ThMADS1
Linum_u_SEP1
Populus_t_PtAGL4
Pisum_s_PEAM6
Medicago_t_MtMADS2
Phaseolus_v_PvSEP1
Glycine_m_GmMADS29
Cucumis_s_CsSEP2
Malus_d_MdMADS1
Malus_d_MdMADS9
Prunus_p_PpMADS7
Fragaria_v_FvMADS_RIN
Rosa_r_MASAKOS1
Vitis_v_VvMADS2
Heuchera_a_HeaSEP1
Petunia_h_PMADS12
Solanum_l_TM29
Petunia_h_FBP5
Antirrhinum_m_DEFH49
Nyssa_s_FBP9
Gerbera_h_GRCD2
Diospyros_k_DkMADS1
Clethra_t_AGL2
Gunnera_m_GmAGL2
FBP9
Passiflora_e_SEP1
Dianthus_c_CMB1
Eucalyptus_g_EGM3
Eucalyptus_g_EGM1
Mangifera_i_MADS1
Passiflora_e_SEP3
Malus_d_MdMADS6
Malus_d_MdMADS3
Taihangia_r_TrSEP1
Solanum_l_SlMADS1
Capsicum_a_CaMADS1
Petunia_h_FBP23
Nicotiana_t_NtMADS4
Petunia_h_FBP9
Davidia_i_FBP9_1
Davidia_i_FBP9_2
Clethra_t_FBP9
Gunnera_m_GmAGL9
SEP4
Capsicum_a_CaMADS_RIN
Solanum_l_SlMADS_RIN
Petunia_h_FBP4
Gentiana_t_GtMADS4
Nyssa_s_AG3
Arabidopsis_t_SEP4
Capsella_r_SEP4
Brassica_r_Brara.F03360.1
Theobroma_c_TcSEP1
Malus_d_MdMADS4
Pachysandra_t_PAteSEP1
Trochodendron_a_MADS3
Nelumbo_n_NEnuSEP1
Nigella_d_NdSEP1
Aquilegia_c_AcoSEP1
Eschscholzia_c_EcAGL2
Epimedium_s_EsAGL2_1
Akebia_t_AktSEP1_1
Nigella_d_NdSEP2
Aquilegia_c_AcoSEP2A
Epimedium_s_EsAGL2_2
Akebia_t_AktSEP1_2
Euptelea_p_EUplSEP1
OsMADS1
Zea_m_ZMM8
Zea_m_ZmM14
Sorghum_b_SbLHS1
Pennisetum_g_PgLHS1
Setaria_i_SiLHS1
Urochloa_m_UmLHS1
Chasmanthium_l_ClLHS1
Danthonia_s_DsLHS1
Eleusine_i_EiLHS1
Eleusine_c_EcLHS1
Aristida_p_AlLHS1
Hordeum_v_HvM7
Avena_s_AsLHS1
Lolium_p_LpMADS9
Lithachne_h_LhLHS1
Dendrocalamus_l_DlMADS13
Leersia_v_LvLHS1
Oryza_s_OsMADS1
Ehrharta_e_EeLHS1
Pharus_l_LHS1
Streptochaeta_a_SaMADS1
OsMADS5
Zea_m_ZMM3
Sorghum_b_SbMADS3
Pennisetum_g_PgMADS1
Oryza_s_OsMADS5
Dendrocalamus_l_DlMADS16
Lolium_p_LpMADS6
OsMADS34
Lolium_p_LpMADS7
Triticum_m_PAP2
Oryza_s_OsMADS34
Zea_m_ZmM24
Zea_m_ZmM31
Elaeis_g_EgAGL2_4
Elaeis_g_EgAGL2_5
Lilium_l_LFMADS3
Houttuynia_c_HcSEP3
Houttuynia_c_HcSEP2
Magnolia_p_MpMADS14
Magnolia_g_MagrAGL2
Nuphar_a_NuadAGL2
Nuphar_p_NupuAGL2.2
Nuphar_p_NupuAGL2.1
Nymphaea_o_NoSEP1
Cabomba_c_SEP1_2
Cabomba_c_SEP1_1
Cabomba_c_SEP1_3
Amborella_t_AMtrAGL2
Outgroups
0.1
Insertion
Deletion
Exonization at the 5' end
Exonization at the 3' end
Pseudoexonization at the 3' end
Intron loss
Gene duplication
